# Functional classification of memory CD8^+^ T cells by CX_3_CR1 expression

**DOI:** 10.1038/ncomms9306

**Published:** 2015-09-25

**Authors:** Jan P. Böttcher, Marc Beyer, Felix Meissner, Zeinab Abdullah, Jil Sander, Bastian Höchst, Sarah Eickhoff, Jan C. Rieckmann, Caroline Russo, Tanja Bauer, Tobias Flecken, Dominik Giesen, Daniel Engel, Steffen Jung, Dirk H. Busch, Ulrike Protzer, Robert Thimme, Matthias Mann, Christian Kurts, Joachim L. Schultze, Wolfgang Kastenmüller, Percy A. Knolle

**Affiliations:** 1 Institute of Experimental Immunology, Universitätsklinikum Bonn, Sigmund-Freud-Street 25, Bonn 53105, Germany; 2 Genomics and Immunoregulation, LIMES-Institute, Universität Bonn, Carl-Troll-Street 31, Bonn 53115, Germany; 3 Max Planck Institute of Biochemistry, Am Klopferspitz 18, München 82152, Germany; 4 Institute of Molecular Immunology and Experimental Oncology, Technische Universität München, Ismaninger Street 22, München 81675, Germany; 5 Institute of Virology, Technische Universität München, Troger Street 30, München 81675, Germany; 6 Clinic for Internal Medicine II, Universitätsklinikum Freiburg, Hugstetter Street 55, Freiburg 79106, Germany; 7 Weizmann Institute of Science, Rehovot 76100, Israel; 8 Institute of Microbiology, Immunology and Hygiene, Technische Universität München, Troger Street 30, München 81675, Germany

## Abstract

Localization of memory CD8^+^ T cells to lymphoid or peripheral tissues is believed to correlate with proliferative capacity or effector function. Here we demonstrate that the fractalkine-receptor/CX_3_CR1 distinguishes memory CD8^+^ T cells with cytotoxic effector function from those with proliferative capacity, independent of tissue-homing properties. CX_3_CR1-based transcriptome and proteome-profiling defines a core signature of memory CD8^+^ T cells with effector function. We find CD62L^hi^CX_3_CR1^+^ memory T cells that reside within lymph nodes. This population shows distinct migration patterns and positioning in proximity to pathogen entry sites. Virus-specific CX_3_CR1^+^ memory CD8^+^ T cells are scarce during chronic infection in humans and mice but increase when infection is controlled spontaneously or by therapeutic intervention. This CX_3_CR1-based functional classification will help to resolve the principles of protective CD8^+^ T-cell memory.

## Abstract (web summary)

The function of memory CD8^+^ T cells is often believed to be directly correlated with their localization in tissues. Here the authors show that CD8^+^ T cells with different proliferative and cytotoxic properties can be distinguished based on their expression of CX3CR1, independently of their tissue localization.

## Graphical abstract

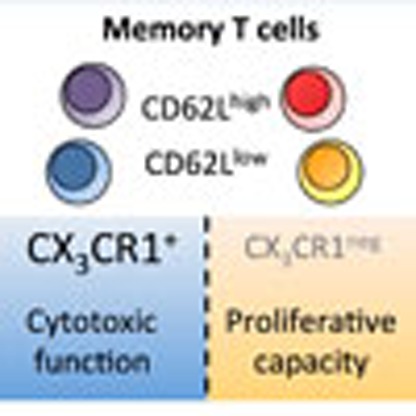

Upon challenge with infectious intracellular microorganisms such as viruses and intracellular bacteria, the immune systems mounts a rapid and commensurate response characterized by an early innate inflammatory response that is followed by generation of pathogen-specific CD8^+^ T-cell immunity. Such CD8^+^ T-cell immunity is important to eliminate or at least contain infection with intracellular pathogens^1^,^2^. Memory CD8^+^ T cells generated in response to the initial pathogen encounter survive in the absence of further antigen-specific stimulation^3^ but also survive during chronic infection and continuous antigen challenge^4^. Memory CD8^+^ T cells provide protection against re-infection with the same pathogen but may also contribute to long-term control of infection if the pathogen cannot be completely eliminated, such as during infection with herpes viruses or hepatitis viruses. Initially, two discrete memory CD8^+^ T-cell populations were characterized by their distinct tissue localization that are believed to be linked to their functionality: central memory T cells (TCM) with proliferative potential that localize to lymphoid tissues and effector memory T cells (TEM) with direct cytotoxic effector functions that reside in peripheral tissues^5^. Consequently, TCM were distinguished from TEM by differential expression of the lymphoid-tissue homing receptors CD62L and CCR7 (ref. 5). Proliferation of memory T cells is required to generate sufficient numbers of effector T cells to control infection, whereas memory T cells with direct cytotoxic effector function are important to provide immediate protection in infected tissues^6^.

However, this strict correlation between memory CD8^+^ T-cell function and their localization was challenged by the finding that T cells with effector functions in the memory T-cell population directly mediate protective immunity^6^ and the discovery of tissue-resident memory T cells (TRM) that possess effector function and have the capacity for self-renewal yet do not recirculate to lymphoid tissues^7^. Furthermore, invasion of lymphoid tissues by bacteria and viruses indicated the necessity of T cells with effector function to be present in lymphoid tissues^8^, which cannot be explained by our current understanding. Rather than looking at bulk T-cell populations that localize to particular tissues, more sophisticated distinction via surface markers is necessary to better understand the mechanisms determining T-cell immunity. Attempts have been made to establish phenotypic markers that predict the functional properties of memory T cells^6^,^9^. Although distinct memory T-cell populations that differ in their functional, proliferative and trafficking characteristics have been recognized^10^,^11^, it has not been investigated whether functionally distinct memory T-cell populations exist among CD62L^+^ TCM in lymph nodes.

Here we report that the expression of the fractalkine receptor CX_3_CR1 discriminates memory CD8^+^ T cells with cytotoxic effector function from those with proliferative potential both in humans and mice. Using CX_3_CR1 together with CD62L as markers, we determine a core gene and protein signature of memory CD8^+^ T cells with cytotoxic effector functions. This allowed us to identify a CX_3_CR1^+^CD62L^hi^ memory T-cell population with direct effector function. This population is stationary in the lymph node and locates to the subcapsular area where pathogens enter. We find low numbers of CX_3_CR1^+^ memory CD8^+^ T cells with effector function in patients suffering from chronic viral infection and high numbers in patients who recovered from viral infection. Also in preclinical models of chronic viral infection, that is, lymphocytic choriomeningitis virus (LCMV) clone 13 infection, numbers of CX_3_CR1^+^ memory CD8^+^ T cells correlate with control of infection and response to immune therapy.

## Results

### CX_3_CR1 expression on memory CD8^+^ T cells

**Figure 1:**
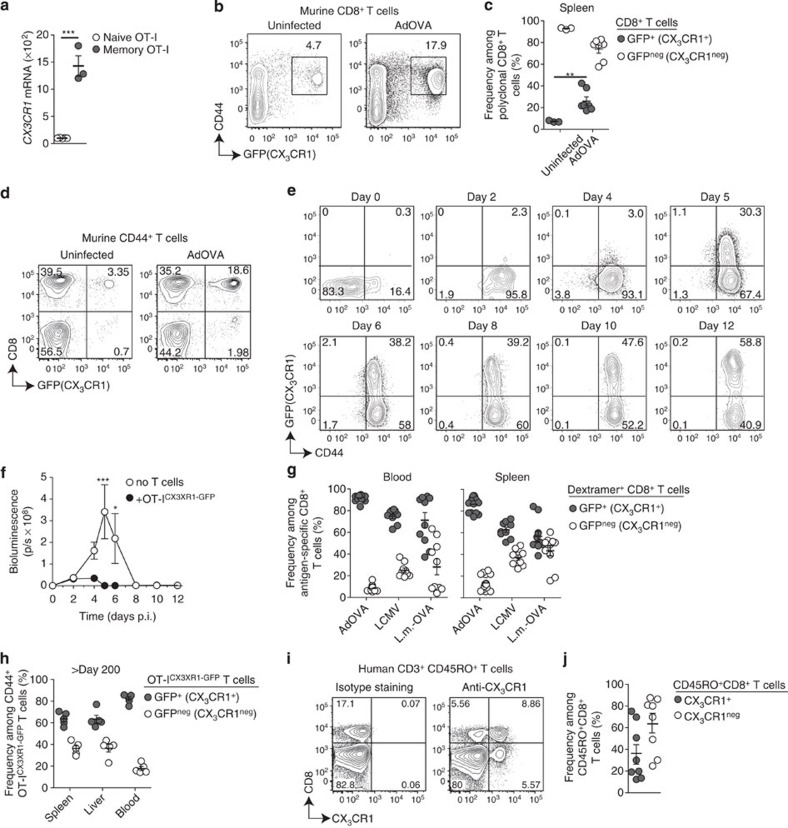
CX_3_CR1 expression on antigen-experienced memory T cells in mice and man. (**a**) Quantification of *Cx*_*3*_*cr1* mRNA levels on CD44^low^ naive OT-I T cells (*n*=5) and CD44^+^ memory OT-I T cells (*n*=3) at >d45 post *L.m.*-OVA infection. ***P<0.001, unpaired *t*-test. (**b**–**d**) Frequencies of GFP^+^ T cells in untreated or AdOVA i.v infected CX_3_CR1^+/GFP^ reporter mice (*n*=7) at 60 d.p.i. isolated from the spleen (**b**,**c**) and the blood (**d**). **P<0.01, unpaired *t*-test. (**e**) C57BL/6 mice that had received 3 × 10^5^ naïve CD44^low^ CD45.1^+^ OT-I^CX3CR1-GFP^ T cells 1 day before were infected with AdOVALUC at day 0 and analysed for GFP(CX_3_CR1)/CD44 expression in OVA-specific CD45.1^+^OT-I^CX3CR1-GFP^ T cells at indicated time points. (**f**) *In vivo* bioluminescence activity of mice (*n*=7) from (**e**) demonstrating efficient OVA-specific T-cell immunity against virus-infected hepatocytes. C57BL/6 mice without adoptive T-cell transfer served as control. *P<0.05 and ***P<0.001, two-way ANOVA. (**g**) CX_3_CR1^+/GFP^ mice were infected with AdOVA (*n*=10), *L.m*.-OVA (*n*=9) or LCMV WE (*n*=10). Frequencies of GFP^+^ (CX_3_CR1^+^) and GFP^neg^ (CX_3_CR1^neg^) cells at 45–60 d.p.i. among splenic OVA-specific CD44^+^CD8^+^ T cells (AdOVA and *L.m.*-OVA infection) or gp33-specific CD44^+^ CD8^+^ T cells (LCMV infection) identified by Dextramer staining. (**h**) Frequencies of GFP^+^ and GFP^neg^ CD45.1^+^ cells at >200 days post AdOVA infection of C57BL/6 wild-type mice (*n*=5) that had received 10^3^ naive CD45.1^+^ OT-I^CX3CR1-GFP^ cells before infection. (**i**) CX_3_CR1 expression in human CD45RO^+^ CD3^+^ T cells isolated from the blood and (**j**) frequency of CX_3_CR1^+^ and CX_3_CR1^neg^ cells among human CD45RO^+^ CD8^+^ T cells (*n*=8). In scatter plots, each circle represents one mouse or patient, fluorescence-activated cell sorting (FACS) plots show representative analysis for one mouse or patient per group. Data are representative for two or three independent experiments (**b**,**d**–**f**, mean and s.d.) or have been pooled from two independent experiments (**c**,**f**–**h**,**j**, mean and s.e.m.). In scatter plots, each circle represents one mouse or a different human donor, FACS plots show representative analysis for one mouse or human donor per group.

We have previously reported a unique murine memory CD8^+^ T-cell population with proliferative potential that is distinct from TCM and is induced by non-professional antigen-presenting cells in the liver but not lymphoid tissues^12^. We reassessed our previously published set of whole-genome transcriptome data utilizing an analysis of variance (ANOVA) model to detect differentially expressed genes as well as self-organizing-map analysis of gene expression profiles to identify genes specific for memory T cells^12^. We found the fractalkine receptor CX_3_CR1 among genes coding for cell surface receptors that were most upregulated within a memory-specific cluster of genes (Fig. 1a and Supplementary Fig. 1A). CX_3_CR1 has been reported to define subsets of myeloid cells, including monocytes and dendritic cells, in the blood and different tissues, such as the gut and lymph nodes^13^,^14^,^15^. So far, CX_3_CR1 expression on a protein level was detected on CD4^+^ T cells residing in the lung or skin^16^,^17^. However, to date, this was not analysed in detail for memory CD8^+^ T cells, although published transcriptome analysis in memory CD8^+^ T cells indicated the expression of the *Cx*_*3*_*cr1* gene^18^,^19^ and CX_3_CR1 was detected on terminally differentiated cytotoxic effector T cells^20^. To analyse the expression pattern of CX_3_CR1 in CD8^+^ T cells during the course of infection and memory formation, we used CX_3_CR1^+/GFP^ reporter mice^13^, in which GFP expression correlated with CX_3_CR1 protein expression (Supplementary Fig. 1B).

In healthy mice, we observed GFP (CX_3_CR1) expression on some antigen-experienced CD44^+^ but not naive CD44^low^ CD8^+^ T cells (Fig. 1b). The frequency of CX_3_CR1-expressing T cells among total CD8^+^ T cells increased after adenoviral infection (d60) (Fig. 1b,c). Among CD44^+^ T cells, CX_3_CR1 expression was most prominent in CD8^+^ T cells (Fig. 1d), prompting us to study CX_3_CR1 expression on antigen-specific memory CD8^+^ T cells in response to infection. After infection with a recombinant adenovirus coding for luciferase and ovalbumin (OVA), which results in hepatocyte infection that can be monitored by *in vivo* bioluminescence measurement^21^, *in vivo* activation of ovalbumin-specific OT-I^CX3CR1-GFP^ T cells was observed at 2 days post infection (d.p.i.) as demonstrated by increased CD44 expression. GFP (CX_3_CR1) expression, however, was not detected before 5 d.p.i. when control over viral infection had been achieved (Fig. 1e), as determined by reduction of *in vivo* bioluminescence (Fig. 1f). The total numbers of GFP^+^ (CX_3_CR1^+^) T cells were highest at 5 d.p.i. and then gradually decreased parallel to the total numbers of antigen-specific CD8^+^ T cells (Supplementary Fig. 1C) consistent with contraction. Antigen-specific GFP^+^ (CX_3_CR1^+^) and GFP^neg^ (CX_3_CR1^neg^) CD8^+^ T cells were found in the lymphoid tissue like in the spleen but also in the blood and liver after viral infection (Supplementary Fig. 1D). The GFP (CX_3_CR1) expression level per CD8^+^ T cell increased during this time by one log (Fig. 1e and Supplementary Fig. 1E). These findings were corroborated studying CX_3_CR1 expression on antigen-specific CD8^+^ T cells generated from the endogenous T-cell repertoire. CX_3_CR1^+^ expression was observed after infection with adenovirus (AdOVA) or *Listeria monocytogenes* (*L.m.-*OVA) on OVA-specific memory CD8^+^ T cells and after LCMV infection on LCMV-gp33-specific memory CD8^+^ T cells (Supplementary Fig. 1F). At 60 d.p.i. of CX_3_CR1^+/GFP^ reporter mice, the majority of CD8^+^ T cells in the blood and spleen, which were specific for OVA (after AdOVA or *L.m.-*OVA infection) or LCMV-gp33, showed GFP (CX_3_CR1) expression (Fig. 1g and Supplementary Fig. 1G). A similar separation in CX_3_CR1^+^ and CX_3_CR1^neg^ populations was observed in memory CD8^+^ T cells generated from 500 adoptively transferred naive OT-I^CX3CR1-GFP^ CD8^+^ T cells (Supplementary Fig. 1H). Even >200 days after AdOVA infection, antigen-specific GFP^+^ (CX_3_CR1^+^) and GFP^neg^ (CX_3_CR1^neg^) memory CD8^+^ T cells were found (Fig. 1h) arguing that both memory T-cell populations are long lived. Importantly, we confirmed CX_3_CR1 expression on T cells in healthy human volunteers. Here, CX_3_CR1 expression was also most prominent on antigen-experienced CD45RO^+^CD8^+^ T cells with numbers varying among healthy individuals (Fig. 1i,j and Supplementary Fig. 1I). Taken together, these data indicated that CX_3_CR1 is expressed in a population of murine and human memory CD8^+^ T cells.

### CX_3_CR1 expression identifies cytotoxic memory CD8^+^ T cells

**Figure 2:**
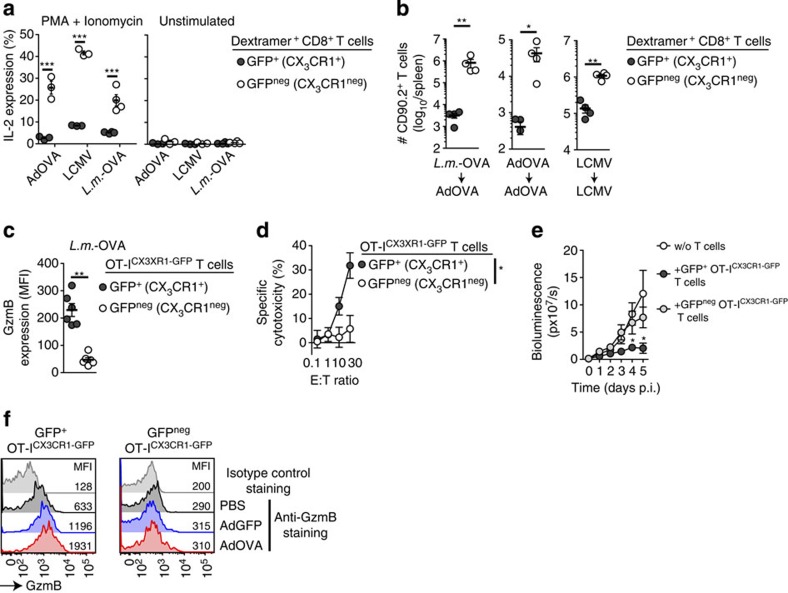
CX_3_CR1 expression separates memory CD8^+^ T cells with distinct functions. (**a**,**b**) CX_3_CR1^+/GFP^ mice were infected with AdOVA (*n*=3), *L.m.*-OVA (*n*=4) or LCMV WE (*n*=3). At 45–60 d.p.i., spleen-derived GFP^+^ (CX_3_CR1^+^) and GFP^neg^ (CX_3_CR1^neg^) memory T cells specific for OVA (after AdOVA and *L.m.*-OVA infection) or for LCMV gp33 were obtained by FACSorting. (**a**) *Ex vivo* IL-2 production after stimulation with PMA/ionomycin. ***P<0.001, unpaired *t*-test. (**b**) Adoptive transfer of sorted OVA-specific or LCMV-specific CD8^+^ T cells (2 × 10^3^) into CD90.1^+^ mice (*n*=4) subsequently infected with AdOVA or LCMV. Determination of CD90.2^+^ T-cell numbers at 8 d.p.i. in the spleen. *P<0.05 and **P<0.01, unpaired *t*-test. (**c**–**e**) Adoptive transfer of FACSsorted naive CD45.1^+^CD44^low^ OT-I^CX3CR1-GFP^ T cells (5 × 10^2^) into CD45.2^+^ mice followed by *L.m.*-OVA infection. (**c**) At 45–60 d.p.i, CD45.1^+^ Memory OT-I^CX3CR1-GFP^ T cells from the spleen were analysed for intracellular GzmB expression (*n*=6). **P<0.01, unpaired *t*-test. (**d**) OVA-specific cytotoxicity of sorted GFP^+^ and GFP^neg^ memory OT-I^CX3CR1-GFP^ T cells directly *ex vivo*. *P<0.05, ANOVA. (**e**) Adoptive transfer of 3 × 10^5^ sorted GFP^+^ or GFP^neg^ memory OT-I^CX3CR1-GFP^ T cells into mice (*n*=6) that were infected with AdOVALUC 4 h before. *In vivo* bioluminescence activity was determined to measure T-cell effector function against virus-infected luciferase-expressing hepatocytes over time. AdOVALUC-infected mice that did not receive T cells served as controls (*n*=6). Data are representative for —two or three independent experiments. *P<0.05, two-way ANOVA. (**f**) Challenge of mice harbouring CD45.1^+^ Memory OT-I^CX3CR1-GFP^ T cells with AdGFP or AdOVA and 6 h later determination of GzmB expression in splenic GFP^+^ and GFP^neg^ memory OT-I^CX3CR1-GFP^ T cells. Data are representative for two independent experiments (**a**,**b**,**d**,**e**, mean and s.d.) or have been pooled from two independent experiments (**c**,**f**, mean and s.e.m.). In scatter plots of **a**–**c**, each dot represents data from one mouse. Fluorescence-activated cell sorting plots shown in **d** are representative for one mouse of a group of three or four mice per experiment.

As GFP (CX_3_CR1) expression separates memory CD8^+^ T cells into two populations (Fig. 1), we addressed the question whether antigen-specific GFP^+^ (CX_3_CR1^+^) and GFP^neg^ (CX_3_CR1^neg^) memory CD8^+^ T cells generated after viral or bacterial infections in CX_3_CR1^+/GFP^ reporter mice had distinct functional properties. The ability to produce the cytokine interleukin-2 (IL-2) is considered a hallmark of those memory T cells that have the potential to proliferate and generate T-cell progeny^22^. We found that GFP^neg^ (CX_3_CR1^neg^) memory CD8^+^ T cells were the main producers of IL-2 upon re-stimulation, whereas GFP^+^ (CX_3_CR1^+^) memory CD8^+^ T cells failed to produce significant amounts of this cytokine (Fig. 2a and Supplementary Fig. 2A). To directly test the proliferative capacity of GFP^+^ (CX_3_CR1^+^) versus GFP^neg^ (CX_3_CR1^neg^) memory CD8^+^ T cells *in vivo*, we adoptively transferred both populations and analysed their numbers after viral or bacterial infections. Transferred GFP^neg^ (CX_3_CR1^neg^) memory CD8^+^ T cells showed vigorous and antigen-specific proliferation upon pathogen challenge, whereas GFP^+^ (CX_3_CR1^+^) memory CD8^+^ T cells showed much less proliferative capacity (Fig. 2b). Similar results were obtained using transgenic OT-I^CX3CR1-GFP^-derived memory CD8^+^ T cells after re-challenge with AdOVA infection (Supplementary Fig. 2B).

Next, we investigated effector functions in GFP^+^ (CX_3_CR1^+^) and GFP^neg^ (CX_3_CR1^neg^) memory CD8^+^ T cells. In contrast to IL-2 expression, only GFP^+^ (CX_3_CR1^+^) memory CD8^+^ T cells at 60 d.p.i. (*L.m.-*OVA) constitutively expressed GzmB (Fig. 2c and Supplementary Fig. 2C), which is a hallmark of T cells with cytotoxic effector function^23^. Consequently, only GFP^+^ (CX_3_CR1^+^) OT-I^CX3CR1-GFP^ memory T cells showed potent cytotoxic effector function directly *ex vivo* (Fig. 2d). After adoptive transfer, GFP^+^ (CX_3_CR1^+^) OT-I^CX3CR1-GFP^ memory T cells but not GFP^neg^ (CX_3_CR1^neg^) T cells conferred immediate *in vivo* protection by rapid control of hepatocyte infection with AdOVALUC (Fig. 2e). In contrast, GFP^neg^ (CX_3_CR1^neg^) T cells did not control infection (Fig. 2e), which is consistent with their lack of cytotoxic functions. Upon pathogen re-challenge, GFP^+^ (CX_3_CR1^+^) OT-I^CX3CR1-GFP^ memory T cells showed further rapid (6 h) increase of GzmB expression. Interestingly, GzmB expression was also triggered by AdGFP infection lacking the cognate antigen but was more pronounced upon AdOVA infection (Fig. 2f), indicating that antigen-specific restimulation was superior to virus-induced inflammation in augmenting GzmB expression. Strikingly, GFP^neg^ T cells remained GzmB negative under such conditions (Fig. 2f), providing a rationale for the failure of this population to confer protective immunity upon transfer (Fig. 2e). Interestingly, no difference in expression of interferon (IFN)-γ was observed between GFP^neg^ and GFP^+^ T cells (Supplementary Fig. 2D). Having established the functional segregation of memory CD8^+^ T cells based on CX_3_CR1 expression, we next reconciled our data with previous work that employed the homing-related molecules CD62L and CCR7 to separate memory CD8^+^ T cells into functionally distinct populations.

### CX_3_CR1 and CD62L identify four memory CD8^+^ T-cell populations

**Figure 3:**
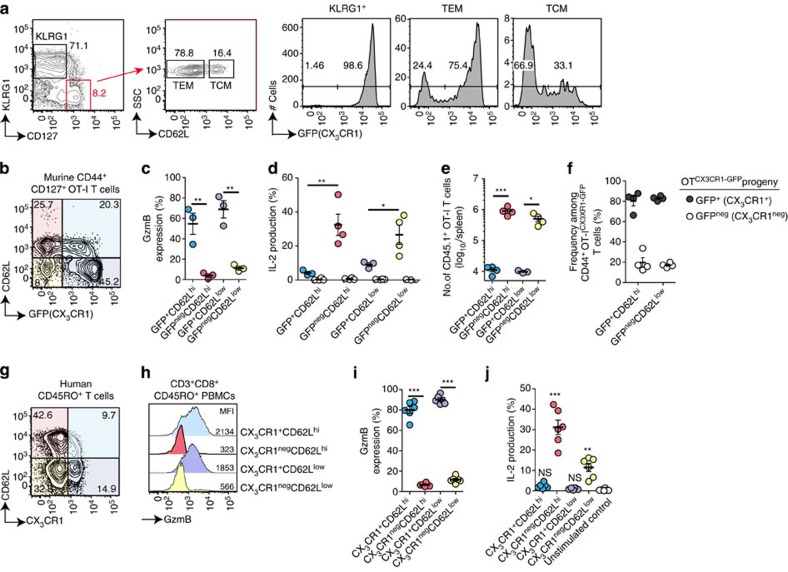
CX_3_CR1 and CD62L expression identifies four distinct memory CD8^+^ T-cell populations. (**a**–**f**) Naive CD45.1^+^ OT-I ^CX3CR1-GFP^ T cells (5 × 10^2^) were adoptively transferred into CD45.2 mice subsequently infected with AdOVA. (**a**) At 60 d.p.i., CX3CR1 expression was determined in CD45.1^+^ OT-I-derived KLRG1^+^ T cells, CD62L^low^CD127^+^CD44^+^ memory T cells (TEM) and CD62L^hi^CD127^+^CD44^+^ memory T cells (TCM) isolated from the spleen. (**b**) Representative analysis of CD62L and GFP (CX_3_CR1) expression in CD127^+^ CD44^+^ memory OT-I^CX3CR1-GFP^ T cells from **a**. (**c**,**d**) Four populations of CD127^+^ CD44^+^ memory OT-I^CX3CR1-GFP^ T cells after AdOVA infection separated by CD62L and GFP (CX_3_CR1) expression levels were (**c**) analysed for GzmB expression or (**d**) sorted and analysed for IL-2 expression after PMA/ionomycine stimulation for 5 h. Unstimulated T cells from each population (empty circles) served as control. *n*=3 or 4 for each group; *P<0.05, **P<0.01, ANOVA. (**e**,**f**) Adoptive transfer of identical numbers (2 × 10^3^ cells) of FACSorted CD45.1^+^ cells from the four populations of memory T cells into CD45.2^+^ mice (*n*=4) that were subsequently infected with AdOVA. (**e**) At 8 d.p.i., total numbers of CD45.1^+^ T cells and (**f**) frequencies of CX_3_CR1^+^ cells among CD45.1^+^ T-cell progeny was determined in the spleen. *P<0.05, ***P<0.001, ANOVA. (**g**) Representative analysis showing CD62L and CX_3_CR1 expression in human CD45RO^+^CD3^+^CD8^+^ PBMCs. (**h**,**i**) Flow cytometric determination of expression of GzmB in the four cell populations stratified by CD62L and CX_3_CR1 expression. ***P<0.001, ANOVA. (**j**) IL-2 expression in FACSorted cells from these four T-cell populations subjected to PMA/ionomycine stimulation for 5 h. **P<0.01 and ***P<0.001, ANOVA. Fluorescence-activated cell sorting (FACS) plots are representative for three (**a**–**c**) or five (**g**,**h**) independent experiments. Data from one of three independent experiments are show in **c**–**f** each dot represents T cells from one mouse. (**i**,**j**) Pooled data for T cells from *n*=6 individual donors.

Memory T cells have been divided into two populations based on the expression of CD62L and CCR7 that allows CD62L^hi^CCR7^+^ TCM to localize to lymphoid tissues, whereas CD62L^low^CCR7^neg^ TEM remain in the blood and peripheral tissues^5^. Although tissue localization correlates with expression of these markers, the separation of functional properties between TCM to generate T-cell progeny and TEM to show cytotoxic effector functions is less stringent^6^,^24^,^25^. This led us to analyse GFP (CX_3_CR1) expression in CD62L^hi^CD127^+^KLRG1^neg^ TCM, CD62L^low^CD127^+^KLRG1^neg^ TEM and KLRG1^+^CD8^+^ T cells derived from naïve OT-I^CX3CR1-GFP^ T cells at 60 days after AdOVA infection. At this time point, all KLRG1^+^ effector-like CD8^+^ T cells showed high GFP (CX_3_CR1) expression (Fig. 3a). However, 25% of TEM did not show GFP (CX_3_CR1) expression (Fig. 3a) and 30% of TCM showed GFP (CX_3_CR1) expression (Fig. 3a). Based on this data we reasoned that CX_3_CR1 in combination with CD62L might enable a highly specific discrimination of memory T-cell subsets with distinct functional properties.

Indeed, using CD62L and CX_3_CR1 in combination, four distinct populations of memory CD8^+^ T cells could be discriminated in mice and healthy humans (Fig. 3b,g). At 60 days after AdOVA infection in mice, GzmB expression was exclusively found in GFP^+^ (CX_3_CR1^+^) memory OT-I^CX3CR1-GFP^ T cells irrespective of their CD62L expression level (Fig. 3c). In contrast, IL-2 production after restimulation was restricted to GFP^neg^ (CX_3_CR1^neg^) memory OT-I^CX3CR1-GFP^ T cells, again irrespective of their CD62L expression levels (Fig. 3d). Along this line, only GFP^neg^ (CX_3_CR1^neg^) CD62L^hi^ and GFP^neg^ (CX_3_CR1^neg^)CD62L^low^ memory OT-I^CX3CR1-GFP^ T cells proliferated after adoptive transfer and AdOVA infection (Fig. 3e). Of note, progeny CD8^+^ T cells were comprised of both, GFP^+^ (CX_3_CR1^+^) and GFP^neg^ (CX_3_CR1^neg^) T cells (Fig. 3f), indicating that CX_3_CR1-expressing CD8^+^ T cells can arise from GFP^neg^ (CX_3_CR1^neg^) memory CD8^+^ T cells during recall responses after viral infection.

Also in the polyclonal repertoire of human CD45RO^+^CD8^+^ T cells, staining for CD62L and CX_3_CR1 identified four cell populations (Fig. 3g). CCR7 expression was observed in all CD62L^hi^ memory T cells and thus did not discriminate CX_3_CR1^+^ from CX_3_CR1^neg^ memory CD8^+^ T cells (Supplementary Fig. 3A). The relative frequencies of these four populations varied between the 18 healthy individuals studied (Supplementary Fig. 3B,C). Only CX_3_CR1^+^CD62L^low^ and CX_3_CR1^+^CD62L^hi^ human memory CD8^+^ T cells expressed GzmB (Fig. 3h,i). Accordingly, CD62L expression levels did not identify GzmB-positive cells among CD45RO^+^ or CD45RA^+^ CD8^+^ T cells, whereas all GzmB-positive CD8^+^ T cells stained positive for CX_3_CR1 (Supplementary Fig. 3D). Furthermore, IL-2 production following restimulation was only observed in CX_3_CR1^neg^ memory CD8^+^ T cells, although we observed more prominent IL-2 production in CX_3_CR1^neg^CD62L^hi^ compared with CX_3_CR1^neg^CD62L^low^ CD8^+^ T cells (Fig. 3i). In mice, several markers, such as CD27, CD28, CD127, and the activation-associated isoform of CD43 (1B11) have been reported to correlate with CD8^+^ T-cell functionality^6^,^9^. As the expression of these markers has not been investigated within CD62L^+^ TCM, especially not in humans, we evaluated their expression in comparison with our CX_3_CR1-based separation on human memory T cells. In human memory T cells, CD43 expression was rather high in CX_3_CR1^+^ CD8^+^ T cells, whereas CD27 showed higher expression on CX_3_CR1^neg^ CD8^+^ T cells (Supplementary Fig. 3E,F) indicating that the co-regulation of CD43 and CD27 on human memory CD8^+^ T cells^9^,^26^ does not allow for clear cut separation with regard to functionality as CX_3_CR1-based separation. CD127 expression levels were slightly higher in CX_3_CR1^neg^ T cells independent of CD62L expression levels (Supplementary Fig. 3E,F) consistent with previous reports on CD127 expression on long-lived central memory T cells^27^,^28^,^29^,^30^. Also, CD28 expression was slightly increased on CX_3_CR1^neg^ T cells (Supplementary Fig. 3E,F) confirming the increased expression found previously on TCM^5^,^31^. Overall, beyond the resolution achieved by CD62L or other T-cell surface markers, CX_3_CR1 expression precisely classifies memory CD8^+^ T cells into two distinct populations in mice and humans independent of their tissue-homing properties, one with cytotoxic effector function but little proliferative capacity, the other with proliferative capacity but no cytotoxic function. To address this notion in an unbiased manner, we decided to apply transcriptomic and proteomic analyses of human memory CD8^+^ T cells.

### Core signature of human CX_3_CR1^+^ memory CD8^+^ T cells

**Figure 4:**
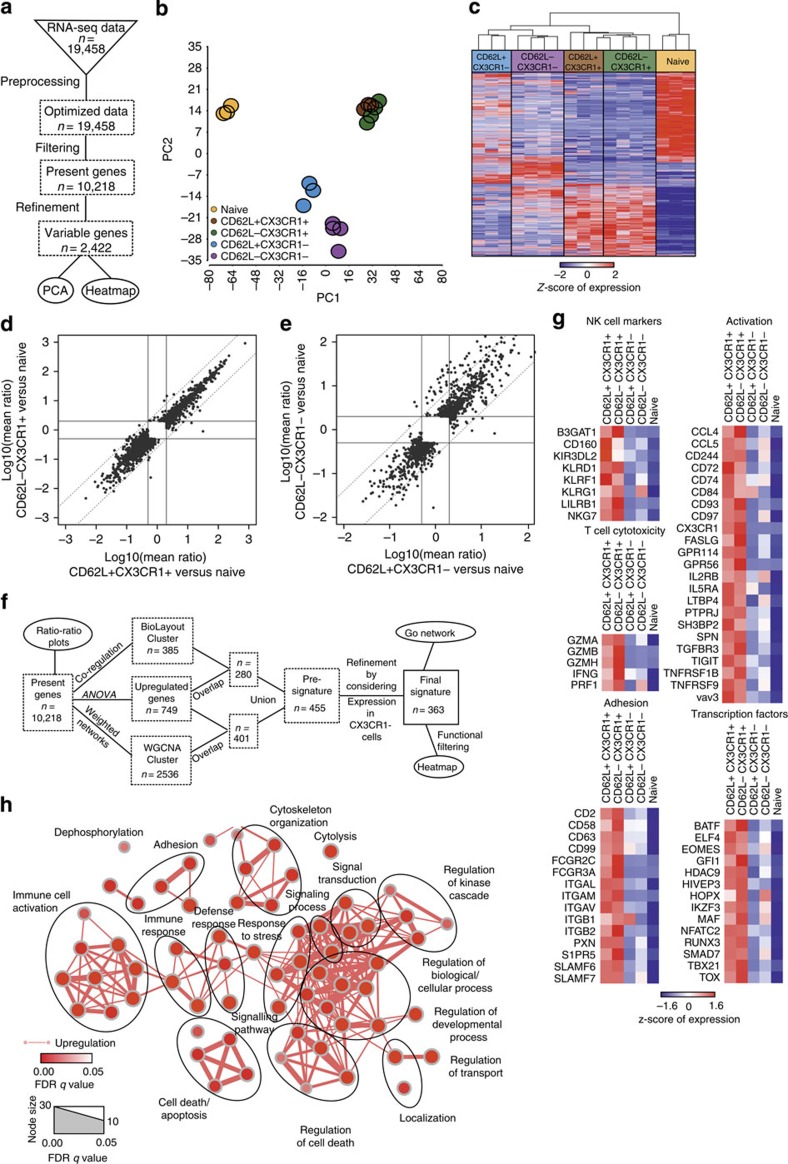
Transcriptome analysis reveals a core signature of human CX_3_CR1-expressing memory CD8^+^ T cells with effector function. (**a**) Scheme describing the workflow for RNA-seq data preprocessing and filtering. (**b**) Principal component analysis (PCA) based on present and variable genes. (**c**) Heat map showing the z-transformed expression values of present and variable genes, coloured from blue to red. (**d**) Ratio–ratio plot of log10-transformed mean ratios of genes that are differentially expressed (fold change (FC) <−2 or >2; FDR-corrected *P*-value <0.05) comparing CD62L^+^ CX3CR1^+^ T cells versus naïve T cells (*x* axis) or CD62L^−^ CX3CR1^+^ T cells versus naïve T cells (*y* axis). (**e**) Ratio–ratio plot of log10-transformed mean ratios of genes being differentially expressed FC <−2 or >2; FDR-corrected *P*-value <0.05) comparing CD62L^+^ CX3CR1^−^ T cells versus naïve T cells (*x* axis) or CD62L^−^ CX3CR1^−^ T cells versus naïve T cells (*y* axis). (**f**) Schema describing the workflow for generating the transcriptome core signature for CX3CR1^+^ T cells. (**g**) Heat map of selected genes out of the 363-gene core signature sorted by defined activity groups. Gene expression values were z-transformed for visualization and are coloured from blue to red. (**h**) Network visualization of Gene Ontology Enrichment Analysis based on the 363 core signature genes using BiNGO and EnrichmentMap. Enriched GO terms are depicted by red nodes, where colour and size represent the corresponding FDR-adjusted enrichment *P*-value (*q*-value). Overlap of genes between nodes is indicated by edge thickness.

To further understand whether CX_3_CR1 expression identifies distinct populations of CD8^+^ T cells, we performed mRNA-sequencing of human naive CD62L^hi^CD45RA^+^CD8^+^ T cells, CX_3_CR1^+^CD62L^hi^ CD45RO^+^CD8^+^ T cells, CX_3_CR1^+^CD62L^low^ CD45RO^+^CD8^+^ T cells, CX_3_CR1^neg^CD62L^hi^CD45RO^+^CD8^+^ T cells and CX_3_CR1^neg^CD62L^low^CD45RO^+^ CD8^+^ T cells, and assessed variable genes by an ANOVA model (Fig. 4a). Principal component analysis of variable genes within the data set clearly revealed one distinct population of CX3CR1^+^CD8^+^ T cells irrespective of their CD62L expression (Fig. 4b). Only in the CX_3_CR1^neg^CD45RO^+^CD8^+^ T cell populations, CD62L discriminated two separate populations, albeit they were more closely related to each other than to naïve CD62L^hi^ CD45RA^+^CD8^+^ T cells and the CX_3_CR1^+^CD45RO^+^CD8^+^ T-cell populations. Hierarchical clustering of variable genes shown as a heat map (Fig. 4c) confirmed these findings as naïve CD45RA^+^CD8^+^ T cells showed the most significant difference to the other four CD45RO^+^ T-cell populations. CX_3_CR1^neg^CD62L^hi^CD8^+^ T cells and CX_3_CR1^neg^CD62L^low^CD8^+^ T cells had distinct gene expression patterns, whereas gene expression patterns of CX_3_CR1^+^CD62L^hi^CD8^+^ T cells and CX_3_CR1^+^CD62L^low^CD8^+^ T cells were almost identical. Consistent with these results and with the usefulness of CX_3_CR1 as marker for functionally distinct T-cell populations, we found that CX_3_CR1 expression was similar to CD43 expression, whereas other markers for memory T cells, such as CD27, CD28 or CD127, were downregulated in CX3CR1^+^ CD8^+^ T cells independent of their CD62L expression (Supplementary Fig. 3G). Focusing on differentially expressed genes as determined by the ANOVA model on present genes directly comparing CD62L^+^ CX3CR1^+^ CD8^+^ T cells or CD62L^−^ CX3CR1^+^ CD8^+^ T cells to naïve T cells and visualizing the results by ratio/ratio plots, further confirmed that CX_3_CR1^+^CD8^+^ T cells had almost identical expression patterns irrespective of CD62L expression levels (Fig. 4d and Supplementary Data 1). In contrast, CD62L^hi^ and CD62L^low^ CD8^+^ T cells lacking CX_3_CR1 showed differential expression of a subset of genes (Fig. 4e and Supplementary Data 1). To determine a core signature for the CX_3_CR1^+^CD45RO^+^CD8^+^ T cells, we performed an analytical approach combining the results of the ANOVA model with either co-regulation analysis or weighted network analysis and subtracted genes also enriched in CX3CR1^−^ T cells to identify differentially expressed genes (Fig. 4f). This approach revealed a set of 363 signature genes (Supplementary Data 2) with high expression in CX3CR1^+^ memory T cells, intermediate to low expression in CX3CR1^−^ memory T cells and low to absent expression in naïve CD8^+^ T cells (Supplementary Fig. 4). We visualized genes of this CX_3_CR1-associated core signature belonging to functional categories including T-cell cytotoxicity markers, NK-cell markers, T-cell activation-associated molecules, adhesion molecules and transcription factors as heat maps of z-transformed expression values (Fig. 4g). This signature included known cytotoxic effector molecules such as FAS-L, perforin and GzmA/B/H, or transcription factors associated with effector function, such as TBX21, BATF, RUNX3 and EOMES, but also other molecules whose relation to CD8^+^ T-cell effector has not been studied yet (Fig. 4g and Supplementary Data 2). Moreover, to better understand which biological processes might be linked to the core signature of CX_3_CR1^+^ CD45RO^+^CD8^+^ T cells, we performed Gene Ontology Enrichment Analysis (GOEA) followed by network visualization (Fig. 4h). The major clusters of biological processes we obtained are compatible with an activated immune cell including Gene Ontology (GO) terms associated with immune cell activation, immune response, defense response, cytolysis, adhesion, response to stress, but also regulation of cell death and cell death/apoptosis. Taken together, the genome-wide analysis of gene expression defined a common gene signature that is shared by human CX_3_CR1^+^CD45RO^+^CD8^+^ T cells irrespective of their CD62L expression that have cytotoxic effector function.

**Figure 5:**
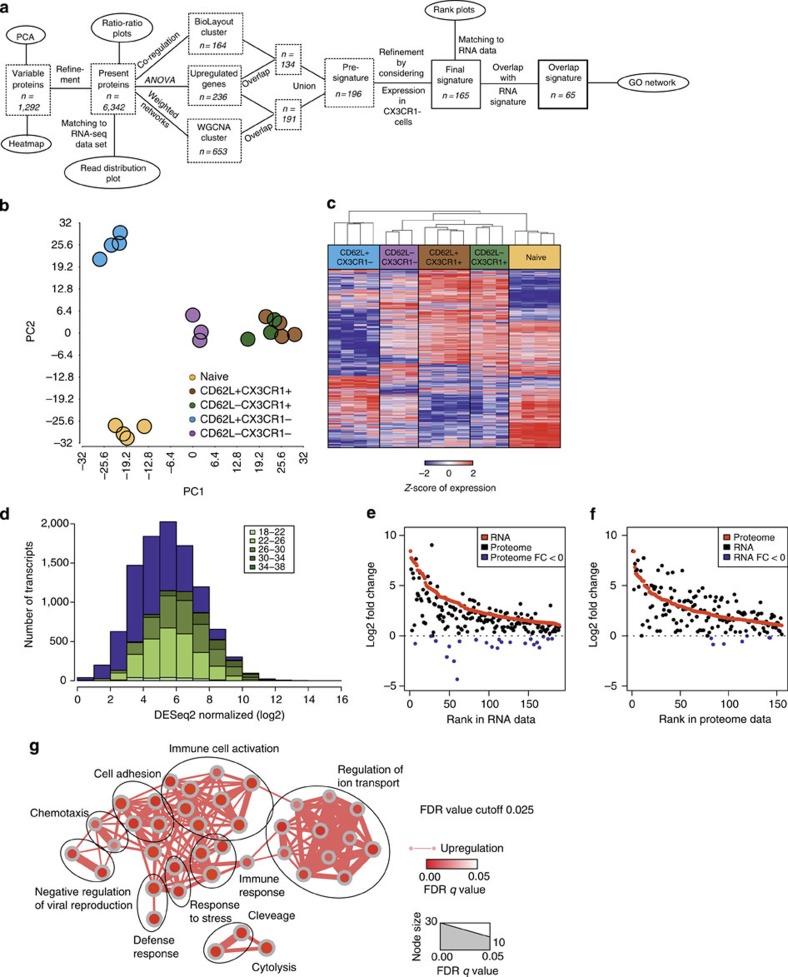
Proteome signature of human CX_3_CR1^+^ memory CD8^+^ T cells with effector function. (**a**) Scheme describing the workflow for analysis of proteome data. (**b**) Principal component analysis (PCA) based on present and variable proteins. (**c**) Heat map showing the z-transformed expression values of present and variable proteins, coloured from blue to red. (**d**) Histogram of normalized RNA-seq expression values of present genes subdivided according to the corresponding log2-transformed protein expression. Violet bars illustrate expression values and amounts of all present transcripts, whereas green-shaded bars represent expression values and amounts of transcripts matched to proteins. (**e**) Fold change rank plot of RNA-seq signature genes (red) with overlay of ranks of the corresponding proteins (black). Proteins having a log2-fold change lower than 0 are marked in blue. (**f**) Fold change rank plot of protein signature genes (red) with overlay of ranks of the corresponding RNA-seq genes (black). Genes having a log2-fold change lower than 0 are marked in blue. (**g**) Network visualization of Gene Ontology Enrichment Analysis using BiNGO and EnrichmentMap based on the 65 genes overlapping between the transcriptome and proteome signatures. Enriched GO terms are depicted by red nodes, where colour and size represent the corresponding FDR-adjusted enrichment *P*-value (*q*-value)<0.025.

Next, we performed proteome analysis of these T-cell populations to establish a global protein profile of the CX3CR1^+^ memory CD8^+^ T cells (Supplementary Data 3 and Supplementary Fig. 5A). Using the identical bioinformatical approach for data analysis (Fig. 5a), we observed a strong similarity between CX_3_CR1^+^CD62L^hi^ and CX_3_CR1^+^ CD62L^low^CD45RO^+^CD8^+^ T-cell populations by principal component analysis, with CX_3_CR1^neg^ CD62L^low^ T cells being most closely related, whereas CX_3_CR1^neg^CD62L^hi^ and naïve CD8^+^ T-cell populations were more distinct (Fig. 5b). This was reflected by hierarchical clustering of variable proteins as both CX_3_CR1^+^CD8^+^ T-cell populations showed close similarities, whereas CX_3_CR1^neg^ CD62L^hi^ and naïve CD8^+^ T cells had clearly different gene expression patterns and CX_3_CR1^neg^ CD62L^low^ T cells revealed a pattern in-between both (Fig. 5c). Visualization of changes in gene or protein expression by volcano plots substantiated that separation of memory CD8^+^ T cells into CX_3_CR1^+^ and CX_3_CR1^neg^ cells is more powerful than separation into CD62L^hi^ and CD62L^low^ cells to identify genetically distinct populations (Supplementary Fig. 5B). We next investigated to which extent candidate molecules from the genome-wide transcriptome core signature were also present in the proteome. Similar to previous findings in other cell types, concordance of expression increased with elevated expression of mRNA and protein^32^ (Fig. 5d). Of the 363 genes being part of the mRNA core signature, 189 were detected by proteome analysis (Supplementary Data 4). Using only those mRNA core signature genes and plotting fold-changes between CX3CR1^+^ and naïve CD8^+^ T cells on mRNA and protein levels revealed that the majority of these cell-type characterizing genes demonstrated concordant gene regulation (Fig. 5e).

To further validate these findings, we next generated a proteome-based signature for CX3CR1^+^ CD8^+^ T cells following the approach visualized in Fig. 4a, which resulted in 165 proteins (Supplementary Data 5). We then used these proteins to annotate the corresponding mRNA data for plotting fold-changes between CX3CR1^+^ and naïve CD8^+^ T cells on protein and mRNA levels (Fig. 5f). Again, except for seven genes, we found concordant gene regulation between proteome and transcriptome suggesting that post-transcriptional regulation is not highly relevant for these core signature genes in CX3CR1^+^ T cells. Next, we determined the overlap of both approaches to reveal a set of 65 signature genes to be highly upregulated in CX3CR1^+^ T cells both on mRNA and protein level (Supplementary Data 6). Functionally, these genes are best described by GOEA with the terms immune cell activation, immune response, defense response, cytolysis, cell adhesion and chemotaxis reminiscent with an activated immune cell (Fig. 5g). Taken together, this mathematical modelling of gene and protein expression demonstrates the value of CX_3_CR1 as a marker to identify distinct populations of memory CD8^+^ T cells that correlate with their functional properties. Furthermore, we establish a core gene and protein signature that identifies memory CD8^+^ T cells with cytotoxic effector functions. Finally, we identify a close similarity between CX_3_CR1-expressing memory T cells irrespective of their CD62L expression.

### CX_3_CR1^+^ CD62L^hi^ CD8^+^ T cells are a resident effector memory population in lymph nodes

**Figure 6:**
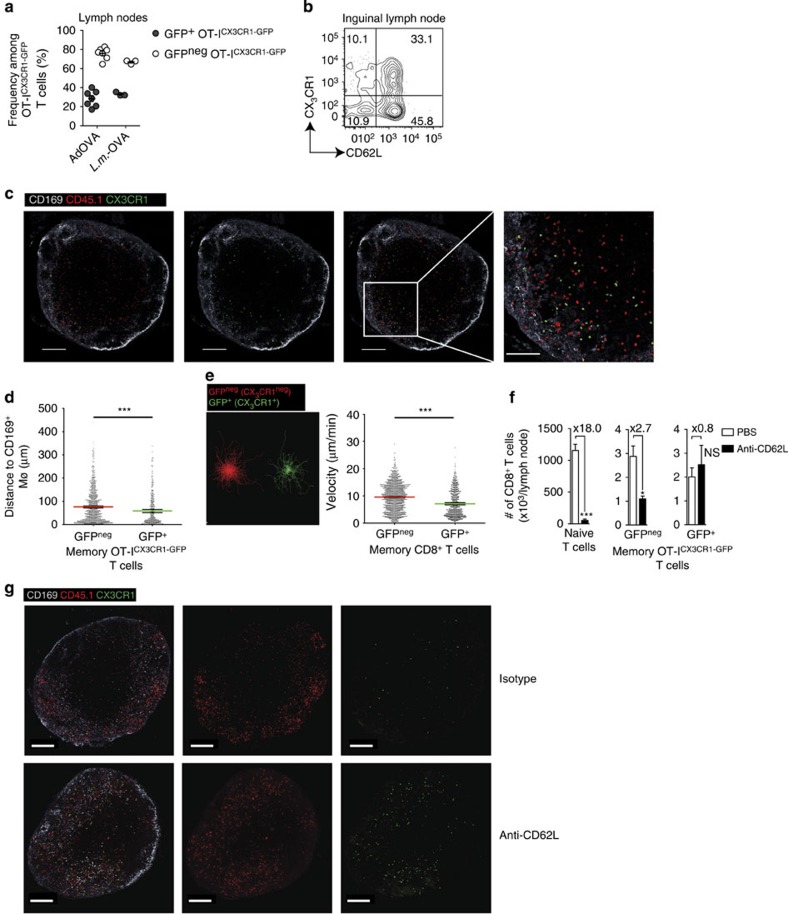
CX_3_CR1 identifies a distinct population of CD8^+^ memory T cells in lymph nodes. (**a**) Quantification of GFP^+^ and GFP^neg^ memory OT-I^CX3CR1-GFP^ T cells within lymph nodes >45 d.p.i. with AdOVA (*n*=7) or *L.m.*-OVA (*n*=3). (**b**) Representative flow cytometric analysis of GFP and CD62L expression in memory OT-I^CX3CR1-GFP^ T cells isolated from lymph nodes. (**c**) Confocal immunofluorescence images of popliteal lymph nodes from a mouse harbouring CX_3_CR1^neg^ and CX_3_CR1^+^ memory OT-I^CX3CR1-GFP^ T cells. Scale bar, 200 μm; zoom 100 μm). (**d**) Relative distance of CX_3_CR1^neg^ and CX_3_CR1^+^ memory CD8^+^ T cells from CD169^+^ MΦ within popliteal lymph nodes. ***P<0.001, *t*-test. (**e**) Track length and average speed comparing CX_3_CR1^neg^ and CX_3_CR1^+^ memory CD8^+^ T cells in the steady state in the interfollicular area over 1 h. ***P<0.001, *t*-test. (**f**,**g**) At 60 days after adoptive transfer of naïve CD45.1^+^ OT-I^CX3CR1-GFP^ T cells (1 × 10^3^) and AdOVA infection, mice were injected daily with anti-CD62L neutralizing antibody (100 μg per mouse i.p.) or PBS over a period of 6 days (*n*=3 per group). (**f**) Quantification of total numbers of endogenous naive CD44^low^CD8^+^ T cells, GFP^neg^CD44^+^ memory OT-I^CX3CR1-GFP^ T cells and GFP^+^CD44^+^ memory OT-I^CX3CR1-GFP^ T cells in inguinal lymph nodes. *P<0.05, ***P<0.001 *t*-test. (**g**) Confocal immunofluorescence images of popliteal lymph nodes at 6 day after anti-CD62L antibody treatment. Scale bar, 200 μm. Data from one of at least two independent experiments are shown.

CX_3_CR1^+^CD62L^hi^CD8^+^ memory T cells closely resembled CX_3_CR1^+^CD62L^low^CD8^+^ memory T cells but were clearly distinct from CX_3_CR1^neg^CD62L^hi^CD8^+^ memory T cells in functional assays (Fig. 2) and transcriptome/proteome analyses (Figs 4 and 5). As indicated by their CD62L expression, we investigated whether CX_3_CR1^+^CD8^+^ memory T cells were present in lymph nodes of CX_3_CR1^+/GFP^ reporter mice. Sixty days after viral or bacterial infection, GFP^+^ (CX_3_CR1^+^) CD62L^hi^CD8^+^ T cells constituted about 20–40% of antigen-specific memory CD8^+^ T cells in lymph nodes (Fig. 6a,b and Supplementary Fig. 6A). Similarly, we detected GFP^+^ (CX_3_CR1^+^) memory CD8^+^ T cells in the white pulp of the spleen (Supplementary Fig. 6B). Although little is known about the heterogeneity of memory T cells present within lymph nodes, two distinct positioning and migration patterns have been described^8^. Therefore, we investigated whether CX_3_CR1^+^ and CX_3_CR1^neg^ CD8^+^ memory T cells differed in their positioning within the lymph node. Confirming our previous results^8^, we found that CD8^+^ memory T cells were not located in the deep paracortex as naïve T cells, but instead were found at the peripheral paracortex and the subcapsular sinus area. This differential positioning of memory versus naïve CD8^+^ T cells was even further pronounced for CX_3_CR1^+^ memory CD8^+^ T cells (Fig. 6c,d). CX_3_CR1^+^CD8^+^ memory T cells had a lower velocity and scanned their environment more slowly than CX_3_CR1^neg^ CD8^+^ memory T cells, as analysed by intravital two-photon microscopy (Fig. 6e and Supplementary Movie 1). We did not observe CX_3_CR1^+^CD8^+^ memory T cells exiting the lymph node, which prompted us to investigate the transit time of memory T-cell populations in the lymph node. We therefore blocked T-cell entry into lymph nodes by anti-CD62L-antibody application (Supplementary Fig. 6B,C). As a result, the numbers of naïve CD8^+^ T cells and to a lesser extent of GFP^neg^ (CX_3_CR1^neg^) memory CD8^+^ T cells declined in lymph nodes (Fig. 6f,g and Supplementary Fig. 6C,D). The numbers of GFP^+^ (CX_3_CR1^+^) memory CD8^+^ T cells, however, remained unaltered over a period of 6 days (Fig. 6f,g and Supplementary Fig. 6B,C). Taken together, CX_3_CR1^+^ memory CD62L^hi^CD8^+^ T cells represent a so far unrecognized lymph node-resident T-cell population positioned in vicinity of CD169^+^ macrophages at the subcapsular sinus in anticipation of pathogens that invade via the lymphatic system. Having established CX_3_CR1 as a marker that identifies memory CD8^+^ T cells with cytotoxic function across tissues, we next aimed to analyse the abundance of such cells in the context of resolved and chronic viral infections.

### Infection control correlates with CX_3_CR1^+^CD8^+^ T-cells

**Figure 7:**
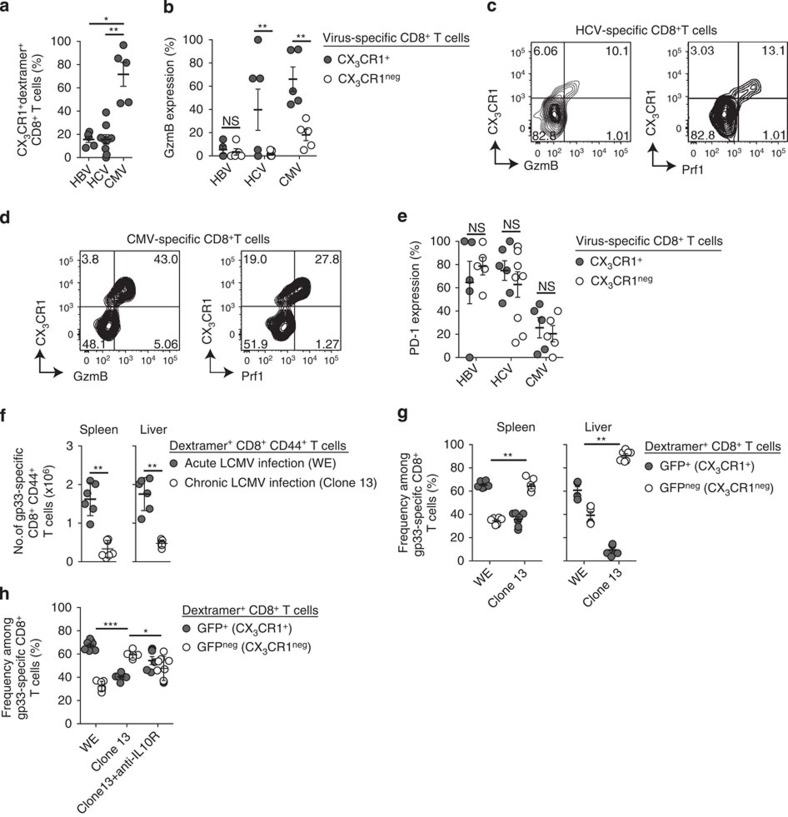
Presence of CX_3_CR1^+^ CD8^+^ T cells during acute resolved and chronic viral infection in man and mouse. (**a**–**e**) CD8^+^ T cells isolated from the blood of human patients chronically infected with HBV or chronically infected with HCV were analysed for virus-specific CD8^+^ T cells identified by tetramer staining. CMV-specific CD8^+^ T cells from the same patients served as control. (**a**) Frequency of virus-specific CX_3_CR1^+^ CD8^+^ T cells. *P<0.05, **P<0.01, *t*-test. (**b**) Flow cytometric analysis of intracellular GzmB expression in CX_3_CR1^+^ and CX_3_CR1^neg^ virus-specific CD8^+^ T cells. **P<0.01, *t*-test. (**c**,**d**) Representative fluorescence-activated cell sorting (FACS) plots showing intracellular GzmB and perforin (Prf1) expression in (**c**) HCV-specific CD8^+^ T cells and (**d**) CMV-specific CD8^+^ T cells. (**e**) Flow cytometric analysis of PD-1 expression in CX_3_CR1^+^ and CX_3_CR1^neg^ virus-specific CD8^+^ T cells. (**f**–**h**) Analysis of LCMV gp33-specific CD8^+^ T cells from CX_3_CR1^+/GFP^ mice 40 days after acute LCMV infection (WE strain; *n*=6) or chronic LCMV infection (Clone 13 strain, *n*=7). (**f**) Quantification of total gp33-specific CD8^+^ T cells in the liver and spleen. **P<0.01, *t*-test. (**g**) Frequency of GFP^+^ (CX_3_CR1^+^) and GFP^neg^ (CX_3_CR1^neg^) cells among gp33-specific CD8^+^ T cells. *P<0.05, **P<0.01, *t*-test. (**h**) Frequency of splenic GFP^+^ (CX_3_CR1^+^) and GFP^neg^ (CX_3_CR1^neg^) gp33-specific CD8^+^ T cells in response to anti-IL10R antibody treatment. *P<0.05, ***P<0.001, ANOVA. **a**,**b**,**e** show data for at least five individual patients in each group, **c**,**d** show representative FACS plots for individual patients. Data in **f**–**h** is pooled from two to three independent experiments, error bars depict s.e.m.

We investigated the frequencies of virus-specific CX_3_CR1^+^CD8^+^ T cells in patients suffering from chronic viral infection, such as chronic Hepatitis B and chronic Hepatitis C. As expected, we could only rarely detect virus-specific CX_3_CR1^+^CD8^+^ T cells in the blood of these patients (Fig. 7a), in contrast to cytomegalovirus (CMV)-specific CD8^+^ T cells from the same donors (Fig. 7a). In some but not all chronic Hepatitis C patients, we found few hepatitis C virus (HCV)-specific CD8^+^ T cells that expressed CX_3_CR1 and also co-expressed GzmB as well as perforin (Fig. 7b,c and Supplementary Fig. 7A), whereas CX_3_CR1^neg^ HCV-specific CD8^+^ T cells did not show GzmB or perforin expression (Fig. 7b,c). We did not detect GzmB or perforin expression in hepatitis B virus (HBV)-specific CD8 T cells in the blood of patients chronically infected with HBV (Fig. 7b). In contrast, we found many CMV-specific CD8^+^ T cells from the same donors who co-expressed CX_3_CR1^+^ and GzmB and perforin (Fig. 7b,d). These results corroborated our findings that CX_3_CR1^+^ T cells have cytotoxic function and directly control viral infections. Of note, PD1 was similarly expressed in CX_3_CR1^+^ and CX_3_CR1^neg^ virus-specific CD8^+^ T cells in chronically infected patients (Fig. 7e), indicating that CX_3_CR1 might more accurately reflect T-cell functionality than PD1 expression. These analyses revealed that virus-specific CX_3_CR1^+^ GzmB^+^ CD8^+^ T cells are found in controlled CMV infection and at low abundance during chronic viral infection in humans.

We next studied CX_3_CR1 expression on virus-specific CD8^+^ T cells during experimental infection with different LCMV clones in mice that is either resolved after acute infection (LCMV WE) or develops into chronic infection (LCMV clone 13; Supplementary Fig. 7D). At 40 d.p.i., we detected significantly more LCMV-gp33-specific CD8^+^ T cells in WE-infected compared with Clone 13-infected CX_3_CR1^+/GFP^ reporter mice in both the spleen and the liver (Fig. 7f). Among those, the frequency of GFP^+^ (CX_3_CR1^+^) CD8^+^ T cells was significantly increased in mice that successfully cleared acute LCMV WE infection compared with clone 13-infected mice (Fig. 7g). In chronically infected mice, GFP^neg^ (CX_3_CR1^neg^) CD8^+^ T cells were abundant among LCMV-specific T cells at 40 d.p.i. (Fig. 7g). Next, we analysed whether successful therapeutic intervention would correlate with re-emergence of cytotoxic CX_3_CR1^+^CD8^+^ T cells. To this end, we treated LCMV Clone13-infected mice with anti-IL-10 receptor antibodies. In line with published data^33^,^34^, this treatment led to a two-log reduction in viral load (Supplementary Fig. 7E) and to an increase in the total numbers of LCMV-specific CD8^+^ T cells (Supplementary Fig. 7F). This increase in LCMV-specific CD8^+^ T cells was followed by an augmented frequency of GFP^+^ (CX_3_CR1^+^) LCMV-specific CD8^+^ T cells (Fig. 7h). Together, these results indicate that virus-specific CX_3_CR1^+^ memory T cells are also present during chronic viral infections albeit at much lower numbers than in resolved infection and that their numbers increase during successful therapeutic intervention.

## Discussion

Discrete memory T-cell populations with complementary functions, executed in distinct anatomic locations, cooperate to mediate immune protection from repeated infection with intracellular pathogens. Lymphoid tissue homing receptors such as CD62L and CCR7 have been employed to distinguish between CD62L^hi^CCR7^+^ TCM that home to lymphoid tissues where they proliferate upon re-challenge and CD62L^low^CCR7^neg^ TEM that remain in the circulation and peripheral tissues where they mount immediate cytotoxic effector function^5^. We identify CX_3_CR1 as the marker that differentiates memory CD8^+^ T cells with direct cytotoxic effector function generated in response to viral or bacterial infections. CX_3_CR1 expression allows their discrimination from memory T cells with proliferative potential. Using CX_3_CR1 together with CD62L as markers, it is possible to stratify memory CD8^+^ T cells in man and mouse into four populations. Genome-wide transcriptome and in-depth proteome analyses provided independent evidence that CX_3_CR1 separates functionally distinct memory CD8^+^ T-cell populations and allowed us to establish for the first time a core gene and protein signature of memory CD8^+^ T cells with cytotoxic effector function. Based on these results, we identify a so far unrecognized memory CX_3_CR1^+^CD62L^hi^ CD8^+^ T-cell population with cytotoxic effector function that localizes to the subcapsular sinus of lymph nodes.

Human and mouse CX_3_CR1^+^ CD8^+^ T cells co-expressed cytotoxic effector molecules (GzmB and perforin) and showed potent cytotoxicity but had no proliferative capacity. Expression of CX_3_CR1 on virus-specific CD8^+^ T cells appeared shortly after clearance of experimental infection, then steadily increased to reach a plateau 2 weeks and was still found up to 200 days after infection. This suggests that early effector T cells, which are generated during the initial phases of the pathogen-specific immune response^35^, do not express CX_3_CR1. However, CX_3_CR1 is expressed shortly thereafter, presumably on both effector T cells and early memory T cells.

After clearance of infection, only long-lived memory CD8^+^ T cells with immediate cytotoxic effector function expressed CX_3_CR1. Some but not all CX_3_CR1^+^ memory T cells also showed expression of KLRG1, consistent with the reported expression of KRLG1 on some memory T cells^6^. CX_3_CR1^+^CD8^+^ T cells found 60 days after infection showed a heterogeneous expression pattern of CD127, indicating that these cells may not exclusively depend on homeostatic survival signals delivered through the IL-7 receptor^27^. Instead CX_3_CR1 itself may promote survival of memory CD8^+^ T cells, similar to the dependence of survival myeloid cells on CX_3_CR1 expression^36^. Expression of CX_3_CR1 has been reported previously for myeloid cells, such as monocytes, macrophages and microglia^37^,^38^, but also for CD4^+^ helper T cells that cause persistent airway inflammation^17^ and for terminally differentiated effector CD8^+^ T cells^20^,^39^. Furthermore, CX_3_CR1 is important for leukocyte migration and adhesion^40^ and recruitment of cytotoxic T cells to sites of inflammation is achieved through CX_3_CL1-expressing cells^20^,^39^. We did not find evidence for changes in the functional phenotype of CX_3_CR1-deficient memory CD8^+^ T cells, indicating that CX_3_CR1-mediated signals are not the cause of direct cytotoxic effector functions in memory T cells.

Notwithstanding, CX_3_CR1 expression accurately distinguished human and mouse memory T cells with direct cytotoxic effector functions from those with proliferative potential. Together with CD62L expression levels, four CD45RO^+^ CD8^+^ T-cell populations can be separated: CX_3_CR1^neg^ CD62L^hi^CD8^+^, CX_3_CR1^neg^CD62L^low^, CX_3_CR1^+^CD62L^hi^ and CX_3_CR1^+^CD62L^low^ CD8^+^ T cells. Transcriptome and proteome analysis of these cell populations revealed that CX_3_CR1 was superior to CD62L to classify distinct memory CD8^+^ T-cell populations based on functional properties. This allowed us to establish a core signature shared by memory CD8^+^ T cells with cytotoxic function independent of their tissue localization. This core signature consists of 363 genes and contains granzymes, perforin, FASL and IFN-γ. The list further entails genes coding for transcription factors associated with effector function such as *Tbx21*, *Batf*, *Nfat*, *Runx3* (refs 41, 42, 43) and surface molecules found on NK cells such as CD57 (B3GAT1), CD160, killer cell lectin receptors and NKG7 (ref. 44) or signalling molecules such as SLAM genes that participate in NK cell function and T -ell differentiation^45^. Many of the core signature genes are also present in the proteome. Some proteins in the core signature, however, are not found in the gene signature such as s100 proteins, which exert alarm functions upon further oxidative modifications^46^. Although expression of these molecules known to be related to cytotoxic effector functions confirms the CX_3_CR1-based core signature, this list will help to refine human immune monitoring to guide immune therapies and initiate research into molecular pathways not yet associated with cytotoxic memory T-cell function.

CX_3_CR1 expression was found on CD62L^low^ as well as CD62L^hi^ memory CD8^+^ T cells and transcriptome and proteome profiles of these two cell populations were almost identical. Based on these analyses both CX_3_CR1^+^CD62^low^ T cells and CX_3_CR1^+^CD62^hi^ memory T cells are best described as memory T cells with effector function. High CD62L expression suggested that some CX_3_CR1^+^memory T cells with effector function could localize to lymph nodes. Indeed, CX_3_CR1^+^ memory CD8^+^ T cells were found in lymph nodes more than 60 days after viral and bacterial infection. These CX_3_CR1^+^CD8^+^ T cells localized to the subcapsular sinus and showed prolonged and continuous interactions with CD169^+^ macrophages, thus taking a strategic position where pathogens entering lymphoid tissues via afferent lymphatics are in first contact with immune cells^8^. It is possible that CX_3_CR1^+^ CD8^+^ T cells in the lymph node may contribute to pathogen-specific immunity through immediate production of IFN-γ, that is required for rapid initiation of immune responses^8^,^47^, after recognizing subcapsular macrophages (cross)presenting microbial antigens. Such rapid induction of IFN-γ may initiate CXCL9-mediated recruitment of CXCR3-expressing memory T cells with proliferative potential^48^ thereby causing timely induction of T-cell expansion upon local pathogen encounter in lymph nodes. They may further help to contain pathogens within this anatomic compartment through killing of infected macrophages^8^. CX_3_CR1^+^CD62L^hi^ CD8^+^ T cells may therefore complement innate lymphoid cells to rapidly mount immune responses in the lymph node^8^. Of note, CX_3_CR1^+^ CD8^+^ T-cell numbers in lymph nodes did not decline despite blockade of lymphocyte re-entry with function-blocking CD62L antibodies, which suggested that these cells remained in lymph nodes for long periods of time. Alternatively, CX_3_CR1^+^ CD8^+^ T cells might be superior to CX_3_CR1^neg^ CD8^+^ T cells in their ability to enter lymph nodes via alternative, CD62L-independent routes. The mechanisms determining positioning and retention of CX_3_CR1^+^ CD8^+^ T cells in lymph nodes and the role of CX_3_CR1^+^ CD8^+^ T cells in lymphoid tissues for induction of pathogen-specific immunity might be assisted by the CX_3_C chemokine interface^39^ but require further investigation.

Another memory T-cell population (TRM) with effector function is resident in epithelial tissues like the skin, gut and lung after local infection^49^,^50^,^51^. TRM develops locally through signalling by IL-15 and transforming growth factor-β from KLRG1^neg^ T cells and are identified by expression of CD103 and CD69 (ref. 52). Both markers are absent from the core signature for memory CD8^+^ T cells with direct cytotoxic function. Furthermore, the core signature of TRM^52^ is distinct from the core signature of CX_3_CR1^+^ CD8^+^ T cells. This suggests that TRM and CX_3_CR1^+^ memory CD8^+^ T cells with cytotoxic effector function develop via separate pathways, which is supported by the expression in CX_3_CR1^+^ CD8^+^ T cells of SMAD7 that regulates transforming growth factor-β-induced signalling^53^. Our finding of CX_3_CR1^neg^CD62L^low^CD8^+^ T cells, however, as memory T cells with proliferative potential that do not recirculate to lymphoid tissues and lack KLRG1 expression, may indicate that these cells are a source for TRM in epithelial tissues. As CX_3_CR1^neg^CD62L^hi^ memory CD8^+^ T cells in lymphoid tissue give rise to CX_3_CR1^+^ T cells with cytotoxic function, it is possible that CX_3_CR1^neg^CD62L^low^CD8^+^ T cells in response to local cues in epithelial tissues give rise to TRM, which provide local tissue protection independently from cytotoxicity by IFN-γ-mediated induction of anti-bacterial and anti-viral genes^54^.

Chronic viral infection develops because of the immune system's inability to eliminate or control acute viral infection. Among the many factors contributing to such failure of immunity, deletion of virus-specific T cells and development of dysfunctional T cells with high expression of co-inhibitory receptors, such as PD1, TIM3 or CTLA4, leading to a dysregulated pattern of gene expression^55^, are believed to be key for chronicity of viral infection^56^,^57^,^58^,^59^. In patients with chronic HCV infection, we found low numbers of circulating virus-specific CX_3_CR1^+^CD8^+^ T cells that co-expressed GzmB and perforin. Only few virus-specific CX_3_CR1^+^CD8^+^ T cells, which lacked GzmB expression, were detected in patients with chronic HBV infection, whereas high numbers of GzmB-expressing CX_3_CR1^+^CD8^+^ T cells were found in CMV-specific CD8^+^ T cells. PD1 expression was similar on both, CX_3_CR1^+^ and CX_3_CR1^neg^ virus-specific CD8^+^ T cells, indicating that CX_3_CR1 expression may help to differentiate between PD1 as marker for recently activated T cells or as marker for dysfunctional T cells. Experimental LCMV infection studies in mice revealed a high ratio of virus-specific CX_3_CR1^+^ to CX_3_CR1^neg^ CD8^+^ T cells in resolved infection, whereas an inverted ratio was observed during chronic LCMV infection. It remains to be determined whether conversion from CX_3_CR1^+^ to CX_3_CR1^neg^ CD8^+^ T cells can occur and causes the observed decrease in CX_3_CR1^+^CD8^+^ T cells in chronic infection. In contrast to the almost identical transcriptome and proteome profiles of CD62L^hi/low^CX_3_CR1^+^CD8^+^ T cells, the profiles of CX_3_CR1^neg^CD62L^low^ CD8^+^ T cells and CX_3_CR1^neg^CD62L^hi^ CD8^+^ T cells were distinct. It is possible that CX_3_CR1^neg^CD62L^low^CD8^+^ T cells comprise a heterogeneous population of cells that contain T cells with proliferative potential that do not relocate to lymphoid tissues but also T cells that were previously CX_3_CR1^+^. Blockade of IL-10 signalling, which is known to strengthen anti-viral immunity during chronic infection^34^, re-invigorated numbers of virus-specific CX_3_CR1^+^CD8^+^ T cells and consequently lead to a two-log reduction in viral load, suggesting that CX_3_CR1^+^CD8^+^ T cells might be sensitive to IL-10-mediated regulation *in vivo*.

Taken together, the use of CX_3_CR1 as marker for identification of memory CD8^+^ T cells with cytotoxic function will help to further our understanding of the principles of T-cell memory and immune protection. Detection of virus-specific CX_3_CR1^+^GzmB^+^CD8^+^ T cells in patients with chronic viral infections suggests ongoing yet attenuated anti-viral immunity^4^. Identification of memory CD8^+^ T cells with immediate cytotoxic function through the core signature defined here will foster the establishment of refined immune monitoring that will allow for improved guidance of immune therapies.

## Methods

### Mice

C57BL/6, CD90.1^+^ C57BL/6, CX3CR1^+/GFP^ (obtained from the Jung Lab), OT-I^CX3CR1-GFP^ (CX3CR^+/GFP^ x T-cell receptor transgenic OT-I), tdTomato OT-I^CX3CR1-GFP^ and CD45.1^+^ OT-I^CX3CR1-GFP^ mice were bred under specific pathogen-free conditions in the central animal facility of the University Hospital Bonn. Mice were kept under specific pathogen-free conditions and *in vivo* experiments were approved by the Local Animal Care Commission of Northrhein Westphalia. Experiments were conducted with sex-matched female or male mice, aged 8–12 weeks at the start of each experiment.

### Generation and analysis of murine memory CD8^+^ T cells

To generate OT-I-derived memory T cells, low numbers (5 × 10^2^ cells) of FACSorted naive CD44^low^ GFP^neg^ CD45.1^+^ OT-I^CX3CR1-GFP^ T cells were adoptively transferred into sex-matched CD45.2^+^ recipient mice. Four hours later, mice were either infected with 5 × 10^3^ colony-forming units *L.m.*-OVA by i.p. injection or infected with 5 × 10^6^ plaque-forming units (PFUs) AdOVA. Memory OT-I cells were identified by the expression of the congenic marker CD45.1 in conjunction with a CD8^+^CD44^+^CD127^+^ phenotype. TCM (CD62L^hi^) and TEM (CD62L^low^) were distinguished by CD62L expression. For functional analysis or adoptive transfer experiments, CD8^+^ T cells were isolated from the spleen by enrichment with autoMACS (untouched CD8^+^ T cell isolation kit, Miltenyi) followed by sorting for the respective markers on a BD Aria III. Experiments were performed with CD44^+^CD127^+^ memory CD8^+^ T cells taken from the spleen at 45–70 d.p.i. if not indicated otherwise.

### Infection models and *in vivo* bioluminescence imaging

*Listeria infection*. Mice were infected i.p. with *Listeria monocytogenes-*expressing OVA (*L.m.-* OVA) acquired from log phase of growth in BHI medium. 5 × 10^3^ colony-forming units were used to generate memory T cells.

*Adenovirus infection*. Recombinant adenovirus expressing either OVA (AdOVA), GFP (AdGFP) or OVA and luciferase as fusion protein (AdOVALUC) were produced in 293 cells and purified by Cs-chlorid gradient ultracentrifugation as described previously^21^. Mice were infected with 5 × 10^6^ PFU AdOVA by intravenous injection (i.v.) for generation of memory T cells. 1 × 10^7^ PFU of AdOVA or AdGFP were used in re-challenge experiments to assess GzmB upregulation by memory T cells. Measurement of bioluminescence in livers of C57BL/6 mice was performed as described previously^21^. In brief, *in vivo* bioluminescence was analysed after intravenous infection with 1 × 10^7^ PFU AdOVALUC using an IVIS 200 system (Caliper LifeSciences) 5 min after i.p. injection of Luciferin (50 mM in PBS, Caliper LifeSciences). Data analysis was performed with Living Image 2.50.1 software (Caliper LifeSciences).

*LCMV infection*. To generate gp-33-specific memory T cells, CX3CR1^+/GFP^ mice were infected intravenously (i.v.) with 2 × 10^4^ PFU of lymphocytic choriomeningitis virus WE-strain (LCMV WE). Mice were infected intravenously with 2 × 10^6^ PFU of LCMV Clone13 or with 2 × 10^4^ PFU of LCMV WE. Titres of virus were determined by plaque assay on Vero cells.

### Isolation of human CD8^+^ T cells

Peripheral blood mononuclear cells (PBMCs) were obtained from patients chronically infected with HBV, HCV, and healthy donors visiting the outpatient clinic of the University Hospital Freiburg. All donors gave written informed consent according to the local ethic committee's of the University hospital Freiburg ruling, federal laws and the Declaration of Helsinki. PBMCs were isolated using density-gradient centrifugation (Pancoll; PAN Biotech, Aidenbach, Germany).

For some experiments, CD8^+^ T cells were isolated by autoMACS separation (Miltenyi) using anti-human CD8 Microbeads (Miltenyi). Virus-specific CD8^+^ T cells were analysed using the following APC-labelled MHC class I-tetrameric complexes: HLA-A*02/HBV core18–27 (FLPSDFFPSV), HLA-A*02/HBV pol455–463 (GLSRYVARL), HLA-A*02/HCV NS31073–1081 (CINGVCWTV), HLA-A*02/HCV NS31406-1415 (KLVALGINAV), HLA-A*02/HCV NS5B2594–2602 (ALYDVVTKL), HLA-B*27/HCV NS5B2841–2849 (ARMILMTHF), HLA-A*02/CMV pp65495-503 (NLVPMVATV). PBMCs were stained with tetramer for 15 min at 37 °C and blocked with murine IgG1 before further staining for surface markers or intracellular cytotoxic proteins.

### Analysis of memory T-cell functions and regulation of T-cell function *in vivo*

To analyse cytokine production, restimulation of FACSorted memory T cells was performed with phorbol myristate acetate (PMA) (5 ng ml^−1^; Sigma-Aldrich) and Ionomycin (200 ng ml^−1^, Sigma) for 4.5 h in the presence of Brefeldin A and Monensin (eBioscience). Restimulation of murine memory T cells from CX3CR1^+/GFP^ mice (CD90.2^+^) was performed in co-culture with CD90.1 splenocytes as feeder cells. Determination of antigen-specific cytotoxicity against peptide-loaded target cells was determined *in vitro* as described^60^. Analysis of intracellular GzmB expression was performed directly after isolation of memory T cells *ex vivo* without further stimulation.

*Blockade of memory T-cell recirculation in vivo*. To block access of circulating CD62L^+^ memory T cells to lymph nodes, mice were injected with 100 μg of anti-CD62L antibody (clone MEL14) or PBS as control i.p. over a period of 6 days before analysis.

*In vivo IL-10R blockade during chronic LCMV infection*. Mice received i.p. five times 250 μg per mouse of IL-10R-specific antibody (clone 1B1.3a; Bio X Cell) or rat IgG isotype control antibody (clone KM1.GL113 Bio X Cell) each third day beginning at 25 d.p.i.

### Flow cytometry and fluorescence-activated cell sorting

Flow cytometric analyses and assessment of mean fluorescence intensity were conducted with a LSR Fortessa (BD Biosciences). Data were analysed using FlowJo software (Tree Star). LIVE/DEAD Fixable Stain kit (Invitrogen) was used to exclude dead cells in all experiments with murine cells, anti-CD16/32 antibody (2.4G2) was used to block unspecific antibody binding via Fc receptors. Human cells were stained in PBS. The following antibodies (purchased from BioLegend or eBioscience) were used for murine samples: CD3 (17A2, dilution 1:200), CD8α (clone 53-6.7, 1:200), CD44 (IM7, 1:300), CD45.1 (A20, 1:200), CD62L (MEL-14, 1:300), CD90.1 (HIS51), 1:400, CD90.2 (HIS51, 1:400), CD127 (ebioSB/199, 1:200), IL-2 (JES6-5H4, 1:200) and IFN-γ (XMG1.2, 1:200). The following antibodies were used for human samples: CD3 (HIT3a, 1:50), CD8 (RPA-T8, 1:10), CD45RA (HI100, 1:25), CD45RO (UCHL1, 1:50), CD62L (DREG-56, 1:50), PD-1 (EH12.2H7, 1:25), CX3CR1 (2A9-1, 1:25), CCR7 (4B12, 1:25) and Perforin (dG9, 1:50). For intracellular staining of cytokines, cells were fixed in 4% PFA and intracellular staining by anti-IFN-γ or anti-IL-2 was performed in Permeabilization Buffer (eBioscience) for 30 min. Staining of Granzyme B (anti-human, cross-reactive with mouse, clone GB11, 1:100) was performed using the Foxp3/Transcription factor staining buffer set from eBioscience. All intracellular stainings in murine cells were done in combination with polyclonal anti-GFP antibody (Life Technologies, Invitrogen; dilution 1:500). Quantification of cell numbers was done with fluorochrome-labelled microbeads (CountBright absolute counting beads, Life Technologies, Invitrogen). Fluorescence-activated cell sorting of splenic naive (CD44^low^GFP^neg^) or memory (CD44^hi^CD127^+^) CD8^+^ T cells was performed with an Aria III cell sorter (BD). Staining with the corresponding isotype antibody served as control. Antigen-specific endogenous memory T cells in mice were identified by staining with fluorochrome-conjugated H-2K^b:SIINFEKL^ dextramers (AdOVA and L.m.-OVA infection; Immudex) or H-2D^b:KAVYNFATC^ dextramers (LCMV infection; Immudex) according to the manufacturer's protocol.

### Immunofluorescence staining

Lymph node and spleens were harvested and fixed using PLP buffer (0.05 M phosphate buffer containing 0.1 M L-lysine (pH 7.4), 2 mg ml^−1^ NaIO_4_ and 10 mg ml^−1^ paraformaldehyde) for 12 h, then dehydrated in 30% sucrose before embedding in OCT freezing media (Sakura Finetek). Thirty-micrometre sections were cut on a CM3050S cryostat (Leica), adhered to Superfrost Plus slides (VWR), stained, mounted with Fluormount G (Southern Biotech) and acquired on a 710 confocal microscope (Carl Zeiss Microimaging). Frozen sections were permeabilized and blocked in 0.1 M Tris (AppliChem) containing 0.3% Triton X-100 (GERBU Biotechnik), 1% FCS (Biochrom AG), 1% GCWFS (Sigma Aldrich) and 1% normal mouse serum (Life Technologies). Serial lymph node sections were prepared, each section was visually inspected using epifluorescent light microscopy, and several representative sections from different lymph node (LN) areas were acquired using confocal microscopy for detailed analysis. The following antibodies were used for staining: anti-CD8 (5H10; Invitrogen), anti-CD169 (c3D6.112; AbDSerotec), ER-TR7 (Santa Cruz Biotechnology), anti-B220 (RA3–6B2; Life Technologies), anti-F4/80 (BM8; BioLegend), anti-CD45.1 (A20; eBioscience). Unconjugated primary antibodies were stained with AF-conjugated secondary antibodies (Life Technologies). For CX_3_CR1 detection, staining with a secondary rabbit anti-GFP (Life Technologies) was used to increase the signal. Primary antibodies were used at a concentration of 1:200, secondary antibodies at a concentration of 1:1,000.

### Intravital two-photon imaging

Mice were anaesthetized with isoflurane (Abbott, Wiesbaden; 2.5% for induction, 1–1.5% for maintenance, vapourized in an 80:20 mixture of O_2_ and air), popliteal LNs were exposed and intravital microscopy was performed using a protocol modified from a previous report^61^. For imaging of td-Tomato and GFP (CX_3_CR1)-transgenic CD8^+^ T cells, we used a Zeiss 780 microscope equipped with a Chameleon laser (Coherent) tuned to 930 nm and a × 20 water dipping lens (numerical aperture=1.0, Zeiss, Jena). The microscope was enclosed in an environmental chamber, in which anaesthetized mice were warmed by heated air, and the surgically exposed lymph node was kept at 36–37 °C with warmed PBS. For dynamic imaging, we recorded a z-stack of 57 μm using 3 μm step size in the interfollicular area and acquired every 40 s. Raw imaging data were processed and analysed with Imaris software (Bitplane).

### Analysis of microarray-based gene expression profiling data

We used a previously generated data set to identify genes associated with memory T cells (GSE63118). Genome Studio (Illumina) was used to profile the raw expression data, where probesets having missing bead types were excluded from the analysis. Processed data of 23 samples were imported into Partek Genomics Suite (PGS) software (v6.6; Partek Inc., log2-transformed and normalized using quantile normalization. Using a background log2-intensity of 6.7886, probesets showing a mean expression lower than this threshold in all five investigated groups were excluded, resulting in 20,515 defined present genes. Their expression values were standardized to a mean of zero and standard deviation of one, and the genes were clustered into 25 groups by using 20,000 training iterations to obtain a self-organizing map. For each condition, the clusters were visualized as heat map based on their eigenvalues, where increased values are shown in red, decreased values in blue and intermediate values in green.

### RNA isolation and purification for RNA-seq analysis

Total RNA was extracted with QIAzol Lysis Reagent (Qiagen) and then purified using the miRNeasy Mini Kit (Qiagen) according to the manufacturers' recommendations. The RNA integrity (RNA Integrity Score ≥6.8) and quantity were determined on the Agilent 2100 Bioanalyzer (Agilent) per the manufacturer's recommendation and subjected to cDNA synthesis.

### cDNA library preparation and RNA sequencing

To generate cDNA libraries, 1,000 pg total RNA were amplified and converted to cDNA using NuGEN's Ovation RNA-Seq kit V2. In brief, the mRNA was reverse transcribed to synthesize the first-strand cDNA by using a combination of random hexamers and a poly-T chimeric primer. Double-stranded DNA is generated by fragmentation of the mRNA template strand using RNA-dependent DNA polymerase. The double-stranded DNA was purified using Agencourt RNAClean XP beads. The DNA is amplified linearly using a SPIA process in which RNase H degrades RNA in DNA/RNA heteroduplex at the 5′-end of the double-stranded cDNA, after which the SPIA primer binds to the cDNA and the polymerase starts replication at the 3′-end of the primer by displacement of the existing forward strand. Finally, random hexamers were used to amplify the second-strand cDNA linearly. Following amplification, 5.0 μg cDNA was fragmented to ∼200 bp using the Covaris S2 and the fragmentation parameters described in the Encore SP Rapid DR Multiplex library preparation protocol (NuGEN). The remainder of the library preparation followed the manufacturer's protocol as described in Encore SP Rapid DR Multiplex System. Paired-end sequencing of bar-coded cDNA libraries at 101 cycles (100 bases each end) was carried out on a HiSeq 1000. The raw sequence data have been deposited to GEO with accession number GSE63147.

### Sample preparation for MS analysis

Cell pellets were washed in PBS and lysed in 6 M Guanidinium chloride (GdmCl), 10 mM HEPES (pH 8) and 10 mM dithiothreitol. Cells were heated for 10 min at 95 °C and sonicated for 15 min at 4 °C (level 5, Bioruptor, Diagenode). Cysteine residues were alkylated in the dark for 30 min with 55 mM iodacetamide. Lysates were diluted 1:3 with 50 mM ammoniumbicarbonate for a proteolytic digest with LysC (1:50, w/w, Wako) for 3 h. Samples were further diluted to 0.6 M GdmCl and digested with trypsin (1:50, w/w, Promega) at room temperature overnight. Buffer exchange was performed on C18 material (Empore, IVA-Analysetechnik). Peptide mixtures were eluted in 80% acteonitrile (ACN) and the organic solvent was removed by centrifugal evaporation. Cleaned peptides were resuspended in 2% ACN, 0.1% trifluoroacetic acid and 0.5% acetic acid.

### LC-MS/MS

Peptides were separated on an EASY-nLC 1000 HPLC system (Thermo Fisher Scientific) coupled online to the Q Exactive mass spectrometer via a nanoelectrospray source (Thermo Fisher Scientific). Peptides were loaded in buffer A (0.5% formic acid) on in house packed columns (75 μm inner diameter, 50 cm length and 1.9 μm C18 particles from Dr Maisch GmbH). Peptides were eluted with a nonlinear 240 min gradient of 5–60% buffer B (80% ACN, 0.5% formic acid) at a flow rate of 250 nl min^−1^ and a column temperature of 50 °C. Operational parameters were real-time monitored by the SprayQC software. The Q Exactive was operated in a data-dependent acquisition mode with a survey scan range of 300–1,700 *m*/*z* and a resolution of 70,000 at *m*/*z* 200. Up to the five most abundant isotope patterns with a charge ≥2 were isolated with a 2.2 Thomson (Th) isolation window and subjected to higher-energy collisional dissociation fragmentation at a normalized collision energy of 25. Fragmentation spectra were acquired with a resolution of 17,500 at *m*/*z* 200. Dynamic exclusion of sequenced peptides was set to 45 s. Thresholds for ion injection time and ion target values were set to 20 ms and 3E6 for the survey scans and 120 ms and 1E5 for the MS/MS scans, respectively. Data were acquired using the Xcalibur software (Thermo Scientific).

### MS data analysis

MaxQuant software (version 1.3.10.18) was used to analyse MS raw files^62^. MS/MS spectra were searched against the human Uniprot FASTA database (Version May 2013, 88'847 entries) and a common contaminants database (247 entries) by the Andromeda search engine. Cysteine carbamidomethylation was applied as fixed and N-terminal acetylation and methionine oxidation as variable modification. Enzyme specificity was set to trypsin with a maximum of two missed cleavages and a minimum peptide length of seven amino acids. A false discovery rate (FDR) of 1% was applied on peptide and protein level. Peptide identification was performed with an allowed initial precursor mass deviation of up to 7 p.p.m. and an allowed fragment mass deviation of 20 p.p.m. Nonlinear retention time alignment of all measured samples was performed in MaxQuant. Peptide identifications were matched across different replicates within a time window of 1 min of the aligned retention times. In addition, single shot runs were match against a T-cell peptide library built from single shot MS measurements of primary human T cells at various activation stages. Protein identification required at least 1 razor peptide. A minimum ratio count of 1 was required for valid quantification events via MaxQuant's Label Free Quantification algorithm (MaxLFQ). Data were filtered for common contaminants and peptides only identified by side modification were excluded from further analysis. In addition, a minimum of three valid quantifications in at least one group of biological replicates was required. Remaining missing values were imputed with random numbers from a normal distribution (mean-shift=1.8; width=0.3), to simulate low abundance values below the noise level.

### Bioinformatics of RNA-seq data

RNA-Seq reads were aligned against the human reference genome hg19 using TopHat v2.0.11. To obtain transcript and gene information, the aligned reads were mapped against the hg19 RefSeq database Release 66 (ref. 63) using Partek Genomics Suite (PGS) software (v6.6; Partek Inc.). Annotated data were normalized using the DESeq2 package within the statistical software R version 3.0.2. In addition, normalized read counts lower than 1 were set equal to 1 to avoid spurious fold changes later on. The genes were filtered to those being present within the data set, defined as having a mean normalized read count larger than 10 in at least one of the investigated groups. Of those, the genes being variable (*P*<0.05) across the data set were visualized via principal component analysis and via a heat map based on z-transformed data using PGS. The list of present genes was then further analysed by three different methods to obtain a core signature for CX3CR1^+^ CD8^+^ T cells. Genes commonly and significantly upregulated in CD62L^+^ CX3CR1^+^ and CD62L^−^ CX3CR1^+^ T cells were identified by using an ANOVA model (FC>2, FDR-corrected *P*-value<0.05). Ratio/ratio plots were used to compare different cell populations on the single-gene level. Clusters of co-regulated genes were determined by applying the Markov Clustering (MCL) algorithm within BioLayout Express^3D^ version 3.2 (ref. 64) with standard criteria on those genes showing a Pearson correlation of at least 0.85. Weighted correlation network analysis was performed in R using a power of 7, a minModuleSize of 30 and a MEDissThres of 0.3 resulting in 20 clusters. For the last two methods, the one cluster representing genes being highly expressed in CX3CR1^+^ and only moderately expressed in CX3CR1^−^ and naïve T cells were chosen. Each of these clusters was then intersected with the list of significantly upregulated genes, and the union of the two intersection lists containing 455 genes was considered as a pre-signature. To refine this signature, genes showing a fold change lower than 1.5 as well as an absolute expression value difference lower than 50 between CX3CR1^+^ and CX3CR1^−^ cells were removed. After finally excluding known polymorph MHCII genes, a signature of 363 genes remained. The distribution of expression values of those genes was displayed separately for all five conditions via boxplots. Sixty-five specific genes were visualized in form of a heat map based on z-transformed data. Finally, all 363 signature genes were linked to prior knowledge by performing GOEA using the Cytoscape^65^ plug-in BiNGO (v2.44) with an FDR threshold of 0.05 to include only significant results. The Cytoscape plugins Enrichment Map (v1.1) with a Jaccard coefficient of 0.25 and an FDR *Q*-value cutoff of 0.025 as well as Word Cloud were used to visualize the GO network.

### Bioinformatics of proteome data

Proteins being variable (*P*<0.05) across the data set were visualized via principal component analysis and via a heat map based on z-transformed data using PGS. To link the RNA-seq expression data to the protein data, the data sets were matched by using the UniProt database. The distribution of normalized RNA-seq expression values of present genes was visualized in the form of a histogram splitted into different protein expression classes. To obtain a core signature for CX_3_CR1^+^ CD8^+^ T cells based on protein data, the same approach as used for the RNA-seq data was applied, harbouring only a few changes in terms of the used parameters. The MCL algorithm in BioLayout Express^3D^ was applied on proteins showing a Pearson correlation of at least 0.8. For weighted correlation network analysis, a power of 18 and a MEDissThres of 0.5 were used. Collating the intersections of each CX_3_CR1^+^ cell representing cluster with the significantly and commonly upregulated proteins resulted in a pre-signature of 192 proteins. After excluding proteins encoded by known polymorph MHCII genes as wells as those proteins having an absolute expression difference of <1e+06 between CX_3_CR1^+^ and CX_3_CR1^−^ cells, a refined signature of 165 proteins remained. Fold change rank plots were used to map their change of expression to their corresponding change in the RNA-seq data set, as well as the same vice versa for the defined RNA-seq signature. Overlapping the transcriptome and proteome signatures resulted in 65 CX_3_CR1^+^-associated proteins, which generated a clearly refined GO network based on the same enrichment parameters as used for the transcriptome-based signature network.

### Statistical analysis

The two-tailed Student's *t*-test was used for the statistical analysis of differences between two groups. Comparison of multiple groups was done using two-way ANOVA.

## Additional information

**Accession codes**: GSE63147.

**How to cite this article:** Böttcher, J. P. *et al.* Functional classification of memory CD8^+^ T cells by CX_3_CR1 expression. *Nat. Commun.* 6:8306 doi: 10.1038/ncomms9306 (2015).

## Supplementary Material

Supplementary Information Supplementary Figures 1-7

Supplementary Data 1 Differentially expressed directly comparing CD62L^+^ CX3CR1^+^, CD62L^-^ CX3CR1^+^, CD62L^-^ CX3CR1^-^, or CD62L^+^ CX3CR1^-^ CD8^+^ T cells to naïve T cells

Supplementary Data 2 Core transcriptome signature of human memory CX3CR1^+^ CD8^+^ T cells

Supplementary Data 3 Protein profile of human memory CX3CR1^+^ CD8^+^ T cells

Supplementary Data 4 Overlap of proteome and transcriptome analysis for core signature

Supplementary Data 5 Core proteome signature of human memory CX3CR1^+^ CD8^+^ T cells

Supplementary Data 6 Core proteome and transcriptome signature of human memory CX3CR1^+^ CD8^+^ T cells

Supplementary Movie 1 Dynamics of CX_3_CR1^+^ memory CD8+ T cell migration in the lymph node. Popliteal LNs of mice harboring memory tdTomato OT-I^CX3CR1-GFP^ CD8^+^ T cells (41 days after immunization with OVA, poly I:C and anti-CD40) were surgically exposed and analyzed by 2-photon microscopy. The movie shows migrational behavior of CX3CR1neg versus CX_3_CR1^+^ memory CD8^+^ T cells.
