# Supplementary material for: Functional classification of memory CD8+ T cells by CX3CR1 expression
Source: Nat Commun. 2015 Sep 25;6:8306. doi: 10.1038/ncomms9306 (PMC4667439; doi:10.1038/ncomms9306)
Supplement: Supplementary Information — Supplementary Figures 1-7 [file ncomms9306-s1.pdf]

Supplementary Figure 1

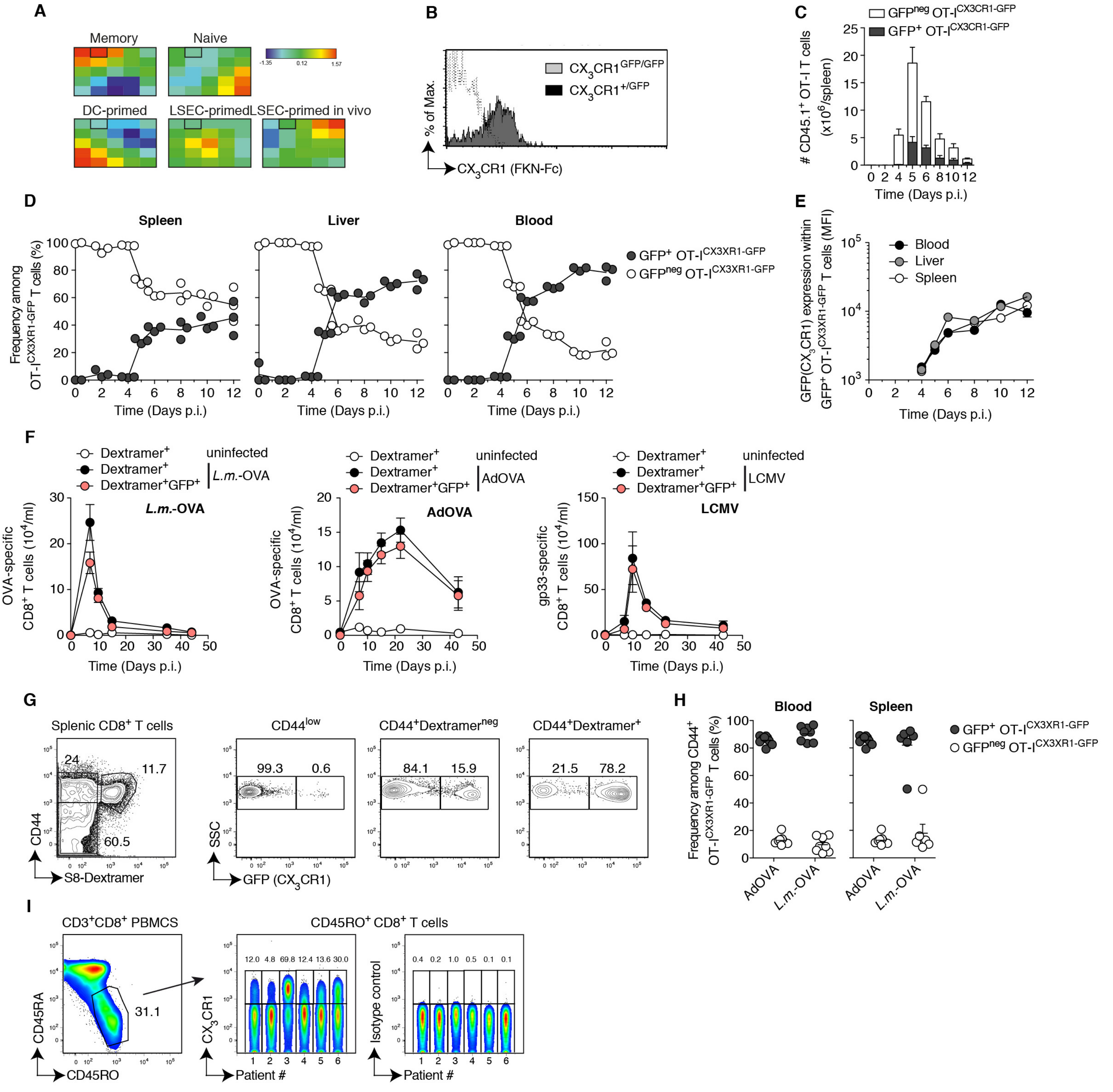

Supplementary Figure 1 *CX_3CR1 expression on $CD8^+$ T cells in man and mouse*
(A) Self-Organizing Map (SOM) clustering based on present genes obtained from gene expression data from memory, naive as well as LSEC-, DC-, and *in vivo* LSEC-primed $CD8^+$ T cells (*1*) was used to identify genes specific for memory $CD8^+$ T cells. The gene cluster containing *CX_3CR1* is marked with a frame. **(B)** GFP^+CD8^+ T cells from CX_3CR1 reporter mice were analyzed for CX_3CR1 protein expression using CX_3CL1-Fc detected with a secondary antibody. **(C-E)** C57BL/6 mice, that had received $3x10^5$ naive $CD44^{low}CD45.1^+$ OT-$I^{CX3CR1\text{-}GFP}$, were infected with AdOVALUC (see Fig. 1 H, I). Time kinetics for **(C)** total numbers of GFP^+ and GFP^{neg} $CD45.1^+$ OT-$I^{CX3CR1\text{-}GFP}$ T cells in spleen, **(D)** frequencies of GFP^+ and GFP^{neg} T cells among $CD45.1^+$ OT-$I^{CX3CR1\text{-}GFP}$ cells in spleen, liver and blood and **(E)** Mean fluorescence intensity (MFI) of the GFP signal in GFP^+ $CD45.1^+$ OT-$I^{CX3CR1\text{-}GFP}$ T cells. **(F)** Time kinetics after infection of $CX_3CR1^{+/GFP}$ mice with AdOVA, *L.m.*-OVA or LCMV (WE strain) for numbers of total and OVA-specific GFP^+CD44^+ $CD8^+$ T cells (after AdOVA and *L.m.*-OVA infection) or LCMV gp33-specific $CD44^+$ $CD8^+$ T cells isolated from blood. OVA-specific or gp33-specific T cells were identified by Dextramer-staining. Uninfected $CX_3CR1^{+/GFP}$ mice served as control. **(G)** Flow cytometric analysis of CX_3CR1 expression in splenic OVA-specific $CD44^+$ $CD8^+$ T cells from $CX_3CR1^{+/GFP}$ mice at d60 after AdOVA infection. OVA-specific T cells were identified by staining with H2-$K^{b:SIINFEKL}$ Dextramers (S8-Dextramer). **(H)** C57BL/6 mice were infected with AdOVA or *L.m.*-OVA after adoptive transfer of 500 FACSsorted naive $CD44^{low}CD8^+$ OT-$I^{CX3CR1\text{-}GFP}$ T cells into $CD45.2^+$ mice. After >45 days, determination of the frequencies of GFP^+ and GFP^{neg} cells among $CD45.1^+$ OT-$I^{CX3CR1\text{-}GFP}$ cells in blood and spleen. **(I)** Flow cytometric analysis of CX_3CR1 expression in $CD3^+CD45RO^+CD8^+$ PBMCs from blood of 6 healthy humans.

Supplementary Figure 2

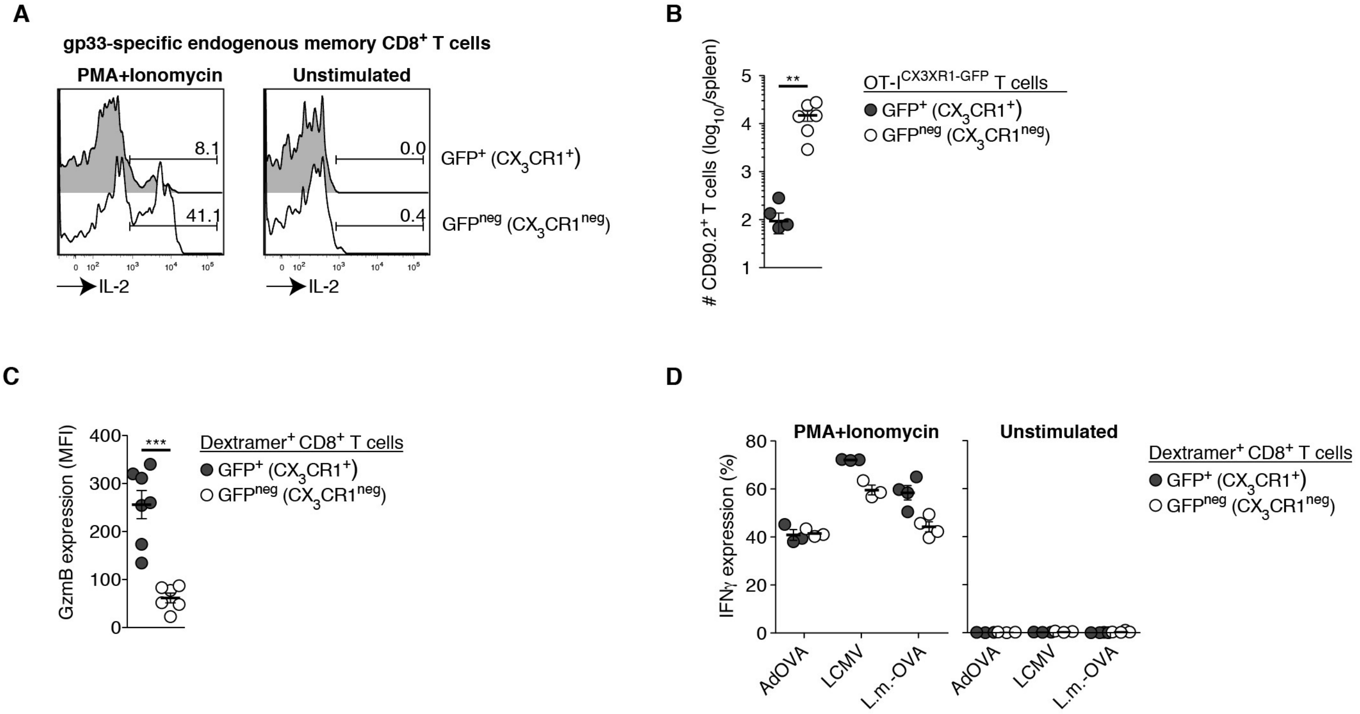

Supplementary Figure 2 *CX_3CR1 expression separates $CD8^+$ T cells with effector function from T cells with proliferative capacity*

(A) Representative analysis of intracellular IL-2 expression in GFP^+ and GFP^{neg} gp33-specific memory T cells isolated from spleen of $CX_3CR1^{+/GFP}$ mice that were infected with LCMV 60 days before. **(B)** $5x10^2$ GFP^+ or GFP^{neg} memory OT-$I^{CX3CR1-GFP}$ T cells ($CD90.2^+$) were adoptively transferred into CD90.1 mice followed by infection with AdOVA. Determination of numbers of $CD90.2^+$ T cells at d8 p.i. in the spleen. **$p<0.01$, t-test. **(C)** Quantification of GzmB expression in (CX_3CR1^+) and GFP^{neg} (CX_3CR1^{neg}) OVA-specific memory T cells from spleen of $CX_3CR1^{+/GFP}$ mice that were infected with *L.m.*-OVA >45 days before. ***$p<0.001$, t-test. **(D)** $CX_3CR1^{+/GFP}$ mice were infected with AdOVA, *L.m.*-OVA or LCMV (WE strain). 45-60 days later, IFNγ production by GFP^+ (CX_3CR1^+) and GFP^{neg} (CX_3CR1^{neg}) memory $CD8^+$ T cells specific for OVA (after AdOVA and *L.m.*-OVA infection) or LCMV gp33 was determined after PMA/Ionomycin stimulation *ex vivo.*

Supplementary Figure 3

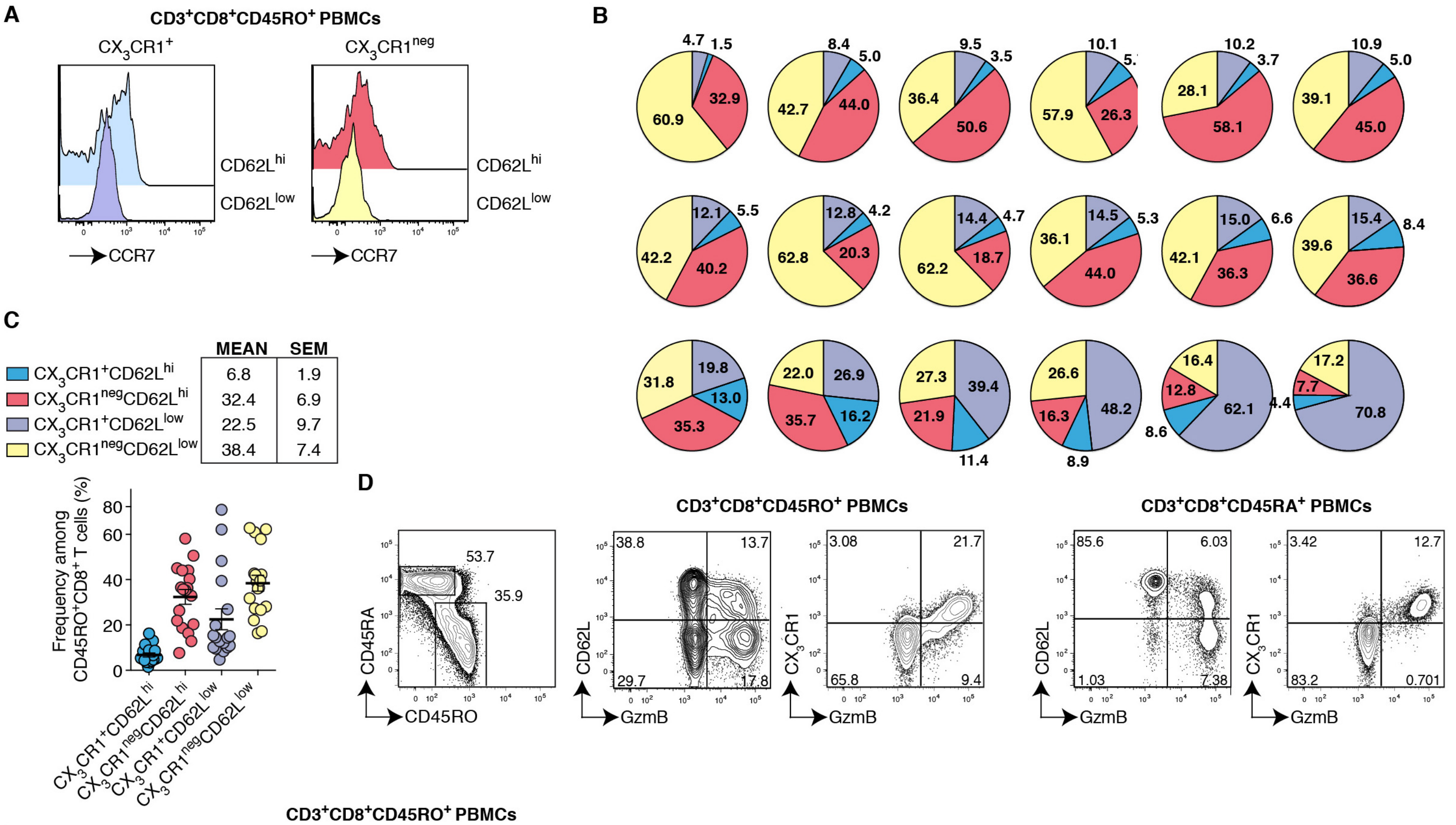

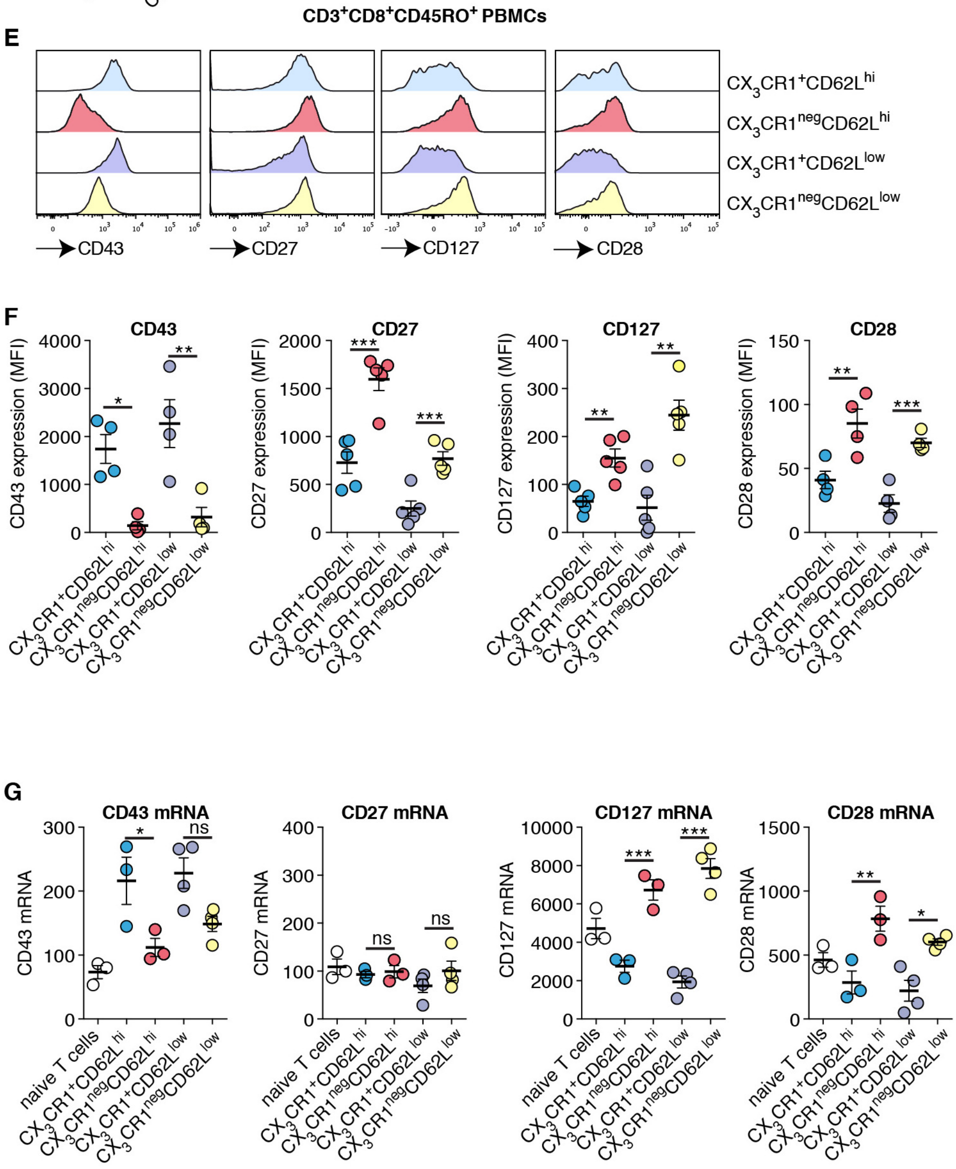

Supplementary Figure 3 *Four populations of human CD45RO+CD8+ memory T cells identified by CX_3CR1 and CD62L*

(A) CCR7 and CD62L surface expression on human CD45RO+ CD8+ memory T cells separated into CX_3CR1^+ and CX_3CR1^{neg} cells. **(B, C)** Frequencies of $CX_3CR1^+CD62L^{hi}$, $CX_3CR1^{neg}CD62L^{hi}$, $CX_3CR1^+CD62L^{low}$ and $CX_3CR1^{neg}CD62L^{low}$ populations within human CD45RO+CD8+ memory T cells. Data are shown for **(B)** each individual patient and **(C)** pooled for all patients with SEM. **(D)** Flow cytometric analysis of human PBMCs showing the power of CD62L and GzmB to identify GzmB+ cells in CD45RO+ CD8+ T cells and CD45RA+ CD8+ T cells. **(E, F)** Flow cytometric analysis CD43, CD27, CD28, and CD127 in combination with CX_3CR1 and CD62L expression on CD3+CD45RO+CD8+ PBMCs from blood of a single individual **(E)** or as cumulative data for 4 healthy human donors **(F)**. **(G)** RNA-seq data for CD43, CD27, CD28, and CD127 in $CX_3CR1^+CD62L^{hi}$, $CX_3CR1^{neg}CD62L^{hi}$, $CX_3CR1^+CD62L^{low}$ and $CX_3CR1^{neg}CD62L^{low}$ human CD45RO+CD8+ memory T cells. Data in (A) and (D) are representative for two independent analyses with at least 4 human patient samples each.

Supplementary Figure 4

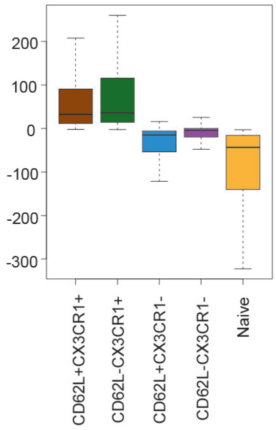

Supplementary Figure 4 *Identification of a core signature for human cytotoxic T cells*

Boxplots representing the distribution of correlation values for each CD8+ T cell subpopulation of the Biolayout cluster used in combination with the ANOVA model to identify the core signature of CX3CR1+ CD8+ T cells.

Supplementary Figure 5

A

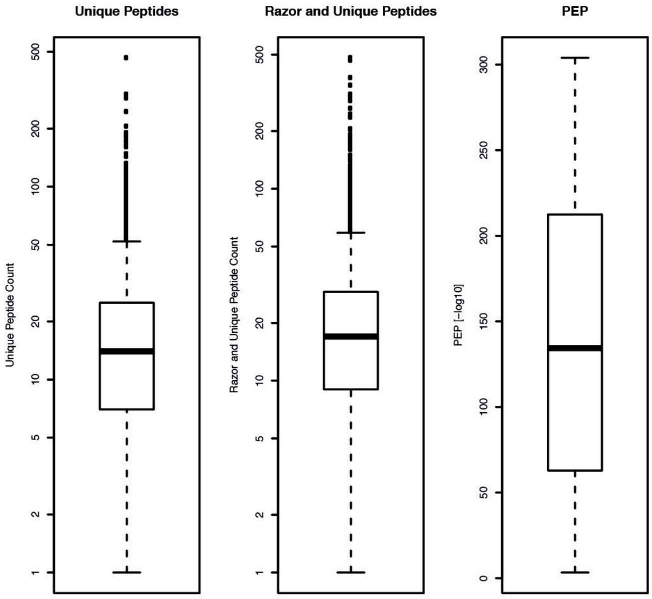

B

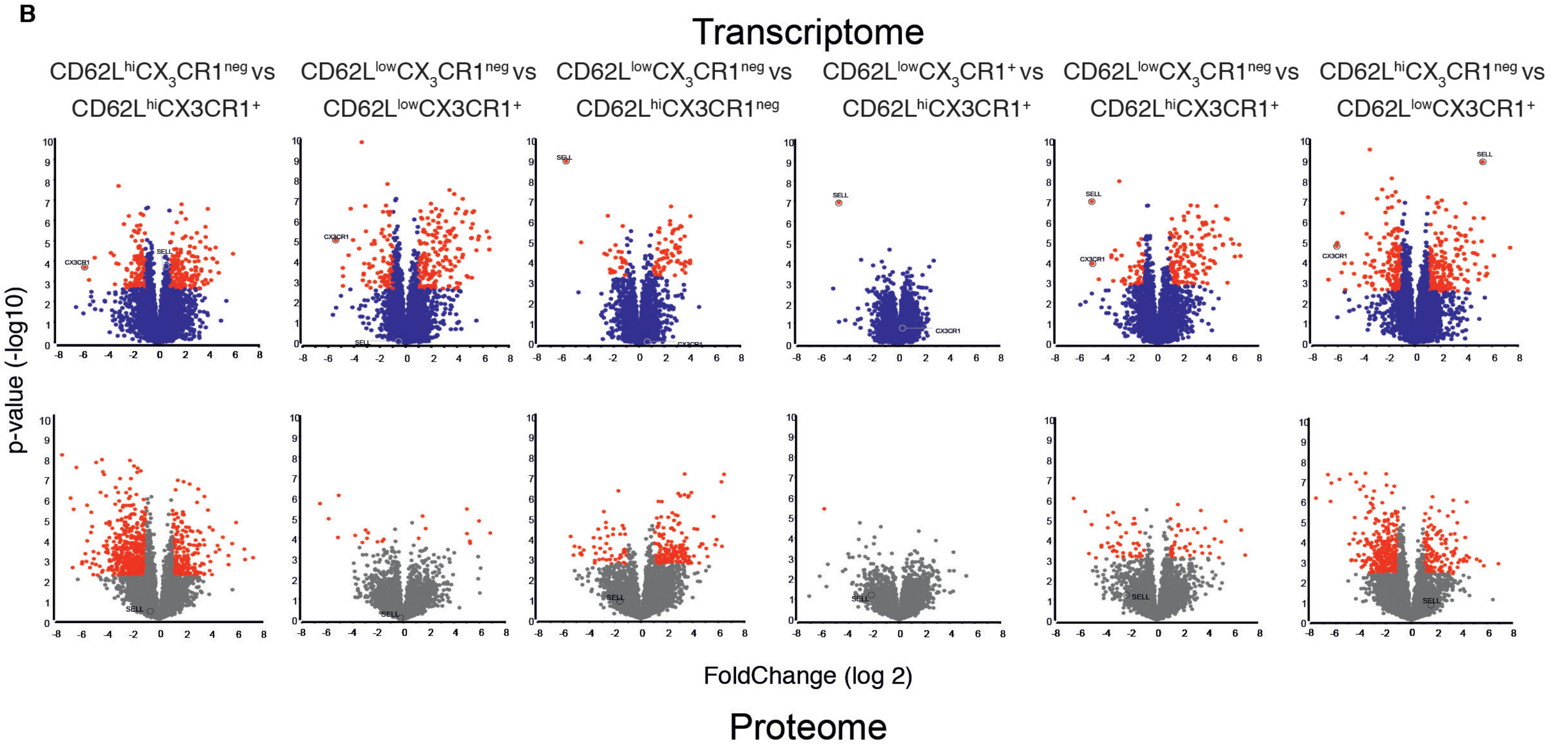

Supplementary Figure 5 *Proteomics identification measures and volcano plot analysis of differences in transcriptome and proteome between memory CD8⁺ T cell populations*

(A) Quality measures of protein group identifications. Numbers of unique peptides per identified protein group, numbers of razor and unique peptides per identified protein group and protein posterior error probability (PEP) for each protein group are shown. Boxplots show median with 5%–95% percentile. All numbers derive from the complete proteome data set including the peptide library. **(B)** Volcano plots displaying log2-fold-change against -log10-p-value of the comparisons of the different CD8⁺ memory T-cell populations as indicated. Upper row: RNA-seq data, Lower row: proteome data

Supplementary Figure 6

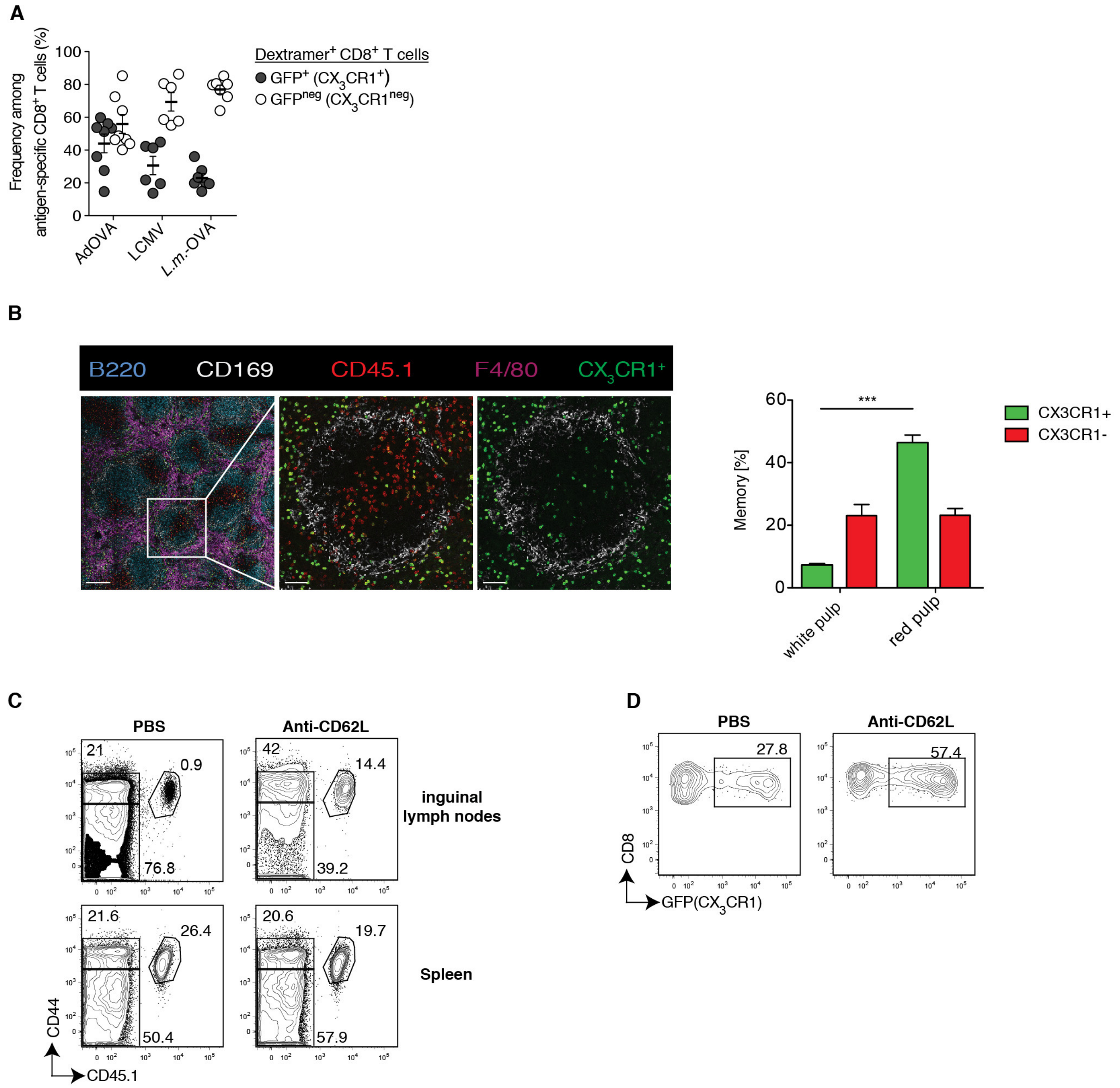

Supplementary Figure 6 *Localization of CX_3CR1 expressing memory $CD8^+$ T cells to lymphoid tissue*

(A) $CX_3CR1^{+/GFP}$ mice were infected with AdOVA, *L.m.*-OVA or LCMV (WE strain). At d45-60 p.i., the frequency of GFP^+ (CX_3CR1^+) and GFP^{neg} (CX_3CR1^{neg}) cells was determined among OVA-specific $CD44^+$ $CD8^+$ T cells (after AdOVA and L.m.-OVA infection) or gp33-specific $CD44^+$ $CD8^+$ T cells (after LCMV infection) identified by Dextramer staining in pooled lymph nodes. **(B)** Detection GFP^+ (CX_3CR1^+) CD45.1+ T cells in spleen. **(C, D)** Mice harboring $CD45.1^+$ memory OT-$I^{CX3CR1\text{-}GFP}$ T cells were injected daily with anti-CD62L neutralizing antibody (100µg/mouse i.p.) or PBS over a period of 6 days. **(C)** Frequency of endogenous (CD45.1 negative) polyclonal naive $CD44^{low}$ $CD8^+$ T cells, $CD44^+$ $CD8^+$ T cells and $CD45.1^+$ memory OT-$I^{CX3CR1\text{-}GFP}$ T cells in inguinal lymph nodes and spleen. **(D)** Frequency of GFP^+ and GFP^{neg} cells among $CD45.1^+$ memory OT-$I^{CX3CR1\text{-}GFP}$ T cells in inguinal lymph nodes. Data is representative for three independent experiments with 3 mice per group

Supplementary Figure 7

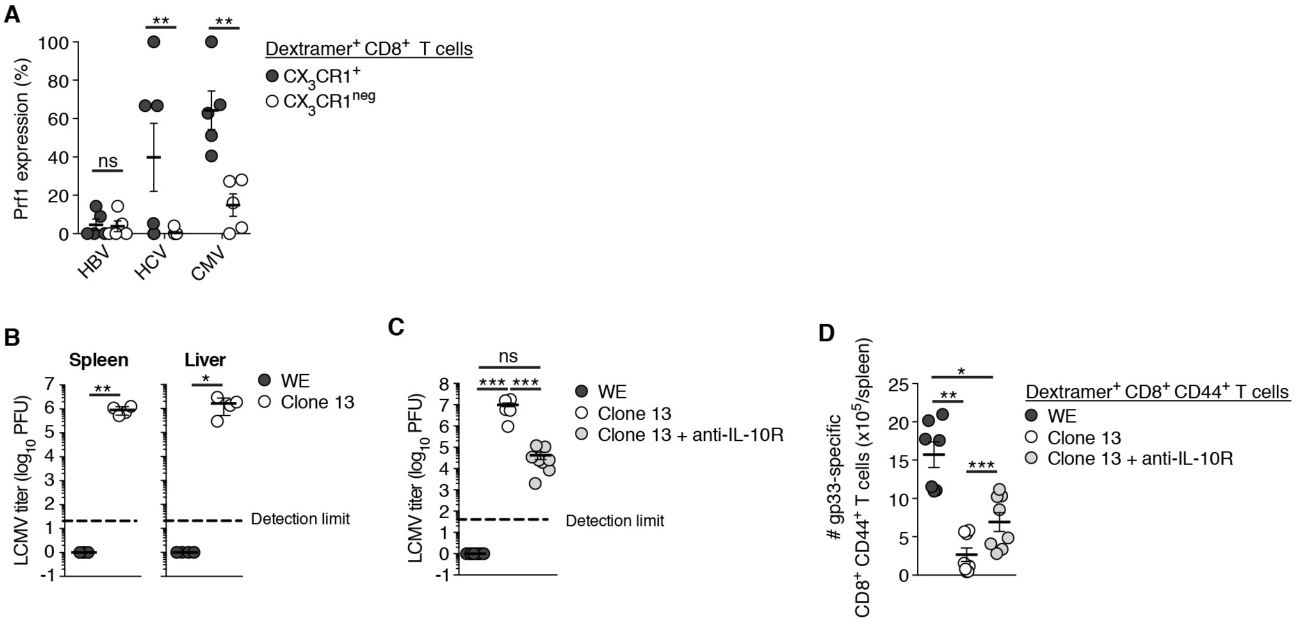

Supplementary Figure 7. *CX_3CR1 identifies cytotoxic virus-specific $CD8^+$ T cells in chronic viral infection* **(A)** Analysis of Perforin (Perf1) expression among CX_3CR1^+ and CX_3CR1^{neg} virus-specific $CD8^+$ T cells isolated from blood of patients chronically infected with HBV or HCV (see Figure 7A, B). CMV-specific $CD8^+$ T cells from the same donors served as control. **p<0.01, t-test. **(B)** Determination of viral titers in liver and spleen of $CX_3CR1^{+/GFP}$ mice 40 days after infection with either LCMV WE or LCMV Clone 13. *p<0.05, **p<0.01, t-test. **(C)** Effect of anti-IL10R treatment on viral titers of LCMV Clone 13-infected $CX_3CR1^{+/GFP}$ mice 40 days after infection. ***p<0.001, ANOVA. **(D)** Quantification of splenic gp33-specific $CD8^+$ T cells of $CX_3CR1^{+/GFP}$ mice 40 days p.i. with LCMV WE, LCMV Clone 13 or after infection with LCMV Clone 13 followed by treatment with anti-IL10R antibody. Each dot represents T cells from one mouse. *p<0.05, **p<0.01***p<0.001, ANOVA.
